# The PLEKHA7–PDZD11 complex regulates the localization of the calcium pump PMCA and calcium handling in cultured cells

**DOI:** 10.1016/j.jbc.2022.102138

**Published:** 2022-06-15

**Authors:** Sophie Sluysmans, Andrea Salmaso, Florian Rouaud, Isabelle Méan, Marisa Brini, Sandra Citi

**Affiliations:** 1 Department of Molecular and Cellular Biology, University of Geneva, Geneva, Switzerland; 2 Department of Biology, University of Padua, Padua, Italy

**Keywords:** pleckstrin homology domain-containing family A, PMCA, PDZD11, PLEKHA7, calcium, AEQ, aequorin, AJ, adherens junction, cDNA, complementary DNA, D-PBS, Dulbecco’s PBS, ER, endoplasmic reticulum, HA, hemagglutinin, IB, immunoblot, IF, immunofluorescence, InsP3, inositol(1,4,5) trisphosphate, KRB, Krebs-Ringer buffer, mCCD, mouse cortical collecting duct, PBM, PDZ-binding motif, PH, pleckstrin homology, PMCA, plasma membrane calcium ATPase

## Abstract

The plasma membrane calcium ATPase (PMCA) extrudes calcium from the cytosol to the extracellular space to terminate calcium-dependent signaling. Although the distribution of PMCA is crucial for its function, the molecular mechanisms that regulate the localization of PMCA isoforms are not well understood. PLEKHA7 is implicated by genetic studies in hypertension and the regulation of calcium handling. PLEKHA7 recruits the small adapter protein PDZD11 to adherens junctions, and together they control the trafficking and localization of plasma membrane associated proteins, including the Menkes copper ATPase. Since PDZD11 binds to the C-terminal domain of b-isoforms of PMCA, PDZD11 and its interactor PLEKHA7 could control the localization and activity of PMCA. Here, we test this hypothesis using cultured cell model systems. We show using immunofluorescence microscopy and a surface biotinylation assay that KO of either PLEKHA7 or PDZD11 in mouse kidney collecting duct epithelial cells results in increased accumulation of endogenous PMCA at lateral cell–cell contacts and PDZ-dependent ectopic apical localization of exogenous PMCA4x/b isoform. In HeLa cells, coexpression of PDZD11 reduces membrane accumulation of overexpressed PMCA4x/b, and analysis of cytosolic calcium transients shows that PDZD11 counteracts calcium extrusion activity of overexpressed PMCA4x/b, but not PMCA4x/a, which lacks the PDZ-binding motif. Moreover, KO of PDZD11 in either endothelial (bEnd.3) or epithelial (mouse kidney collecting duct) cells increases the rate of calcium extrusion. Collectively, these results suggest that the PLEKHA7–PDZD11 complex modulates calcium homeostasis by regulating the localization of PMCA.

Pleckstrin homology (PH) domain containing family A member 7 (PLEKHA7), a cytoplasmic component of adherens junctions (AJs) (1, 2), recruits the small adapter protein PDZ domain-containing protein 11 (PDZD11) to junctions (3, 4). The N-terminal region of PDZD11 binds to the N-terminal tandem of WW domain of PLEKHA7 and related WW-PLEKHA proteins (3, 5, 6, 7). In a complex with PDZD11, PLEKHA7 and other WW-PLEKHAs modulate the trafficking, plasma membrane localization, and function of transmembrane proteins that interact with PDZD11 through their PDZ-binding motif, such as the immunoglobulin-like (Ig-like) adhesion proteins nectins (3) and the Menkes copper ATPase (7, 8). In addition, PLEKHA7 binds to the cytoplasmic C-terminal region of the transmembrane protein tetraspanin-33 through its WW domains, and this interaction is enhanced by PDZD11 to control the localization of the transmembrane metalloprotease ADAM10 (5, 9). Genome-wide association studies in human cohorts implicate PLEKHA7 in blood pressure and hypertension (10, 11, 12). A mechanism through which PLEKHA7 could modulate blood pressure is the regulation of calcium homeostasis, since depletion of the PLEKHA7 homolog heart adapter protein 1 (Hadp1) in zebrafish alters cardiac contractility (13), and PLEKHA7 mutant rats exhibit attenuated hypertension in response to a high salt diet (14), correlating in both cases with altered calcium handling. However, the mechanisms through which PLEKHA7 regulates calcium homeostasis are unknown.

The plasma membrane calcium ATPase (PMCA) is an essential transmembrane pump, which controls cellular calcium levels by extruding Ca^2+^ from the cytoplasm toward the extracellular space (15). The localization of PMCA isoforms to distinct membrane domains and microdomains is important for cellular signaling (16, 17, 18, 19). In mammals, four genes encode PMCA isoforms 1 to 4, with additional isoform diversity generated by alternative mRNA splicing (20, 21). PMCA4 is ubiquitously expressed and is involved in regulation of systemic blood pressure and arterial calcium handling (22, 23). The alternative splicing in the C-terminal coding region of the four PMCA isoforms generates either a truncated or a full-length C-terminal cytosolic tail of the protein, resulting in either “a” or “b” variants, respectively (24). The last four amino acids of “b” variants encode a PDZ-binding motif (PBM) (25), which is missing in “a” variants. The PBM was shown to bind to the small adapter protein PDZD11, also known as PISP (Plasma membrane calcium ATPase-Interacting Single-PDZ protein) (26). However, the role of PDZD11 in either PMCA localization or calcium handling is not known.

Since PLEKHA7 and PDZD11 play a role in the trafficking, localization, and function of interacting transmembrane proteins (3, 7, 9), we hypothesized that they may also regulate PMCA localization and function. To test this hypothesis, we examined the localization of endogenous PMCA and exogenous PMCA4x/b in polarized kidney epithelial cells WT or KO for either PLEKHA7 or PDZD11. We also studied the activity of PMCA4x/b and PMCA4x/a in an exogenous reconstituted system of HeLa cells with the coexpression of either PDZD11 or PLEKHA7. Finally, we analyzed calcium extrusion in endothelial (bEnd.3) and epithelial (mouse cortical collecting duct [mCCD]) cells, either WT or KO for PDZD11. Our results show that PLEKHA7 and PDZD11 modulate the accumulation of endogenous PMCA at lateral cell–cell contacts and exclude it from apical epithelial junctions. Moreover, PDZD11 reduces cytosolic calcium extrusion and PMCA targeting to the plasma membrane in an exogenous reconstituted system in HeLa cells. Finally, the KO of PDZD11 in bEnd.3 and mCCD cells results in increased calcium extrusion.

## Results

### PLEKHA7 and PDZD11 regulate the accumulation of endogenous PMCA at cell–cell contacts

**Figure 1:**
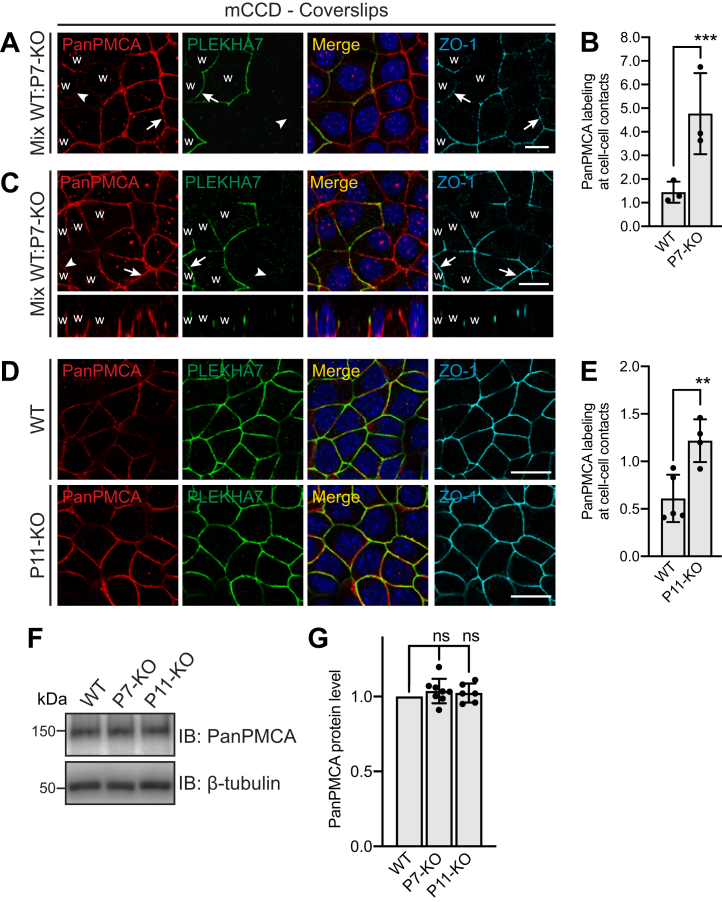
**PMCA accumulation at cell–cell contacts is increased in epithelial cells KO for either PLEKHA7 or PDZD11.***A*–*C*, IF microscopy (*A*, *C*) of endogenous PMCA (PanPMCA antibody), PLEKHA7 (to distinguish WT and KO cells), and ZO-1 (junctional reference marker) in mixed cultures of WT (w) and PLEKHA7-KO (P7-KO) mouse kidney collecting duct (mCCD) cells. Merge images show colocalization between PMCA and PLEKHA7 labeling. *Arrows* indicate labeling; *arrowheads* indicate lower or undetectable labeling. Z sections taken at the horizontal middle position of the respective XY view are shown below square panels in (*C*). The scale bars represent 20 μm. (*B)*, shows quantification of junctional labeling for PMCA at cell–cell contacts in WT and P7-KO cells, ratioed to ZO-1. *Dots* show replicates (n = 3) and *bars* represent mean ± SD. Ratio paired *t* test (∗∗∗*p* < 0.001). *D* and *E*, IF microscopy (*D*) and quantification (*E*) of endogenous PMCA labeling in either WT or PDZD11-KO (P11-KO) mCCD cells. Merge images show colocalization between PMCA and PLEKHA7 labeling. The scale bars represent 20 μm. Quantification (*E*) of junctional labeling for PMCA in WT and P11-KO cells, using ZO-1 as cell–cell contact reference. *Dots* show replicates (n=4–5) and *bars* represent mean ± SD. Unpaired *t* test (∗∗*p* < 0.01). *F* and *G*, IB analysis (*F*) and quantification (*G*) of endogenous PMCA (Pan-PMCA antibody) in lysates of either WT or PLEKHA7-KO (P7-KO) or PDZD11-KO (P11-KO) mCCD cells. Quantification (*G*) of PMCA signal, normalized to ß-tubulin, in PLEKHA7-KO and PDZD11-KO mCCD cells, relative to the WT. *Dots* show replicates (n=6–9) and *bars* represent mean ± SD. Mixed-effects analysis with post hoc Dunnett’s test (ns, not significant). IF, immunofluorescence; mCCD, mouse cortical collecting duct; PMCA, plasma membrane calcium ATPase.

To determine whether PLEKHA7 and PDZD11 regulate PMCA localization, we analyzed by immunofluorescence (IF) microscopy the localization of PMCA in epithelial cells from the collecting duct of mouse kidney (mCCD), either WT or KO for either PLEKHA7 or PDZD11. We chose polarized epithelial cells for localization studies because they display spatially defined apical, lateral, and basal domains of the plasma membrane and are thus ideally suited to investigate subtle changes in the localization of membrane proteins. Mixed cultures of WT and PLEKHA7-KO cells were labeled with antibodies against PLEKHA7, to distinguish WT from KO cells, and with antibodies against ZO-1, to identify apical junctions (Fig. 1*A*). In XY images, PMCA was accumulated at the cell periphery, and the intensity of PMCA labeling at cell–cell contacts was significantly higher in PLEKHA7-KO cells compared to neighboring WT cells (*arrows* and *arrowheads* in Fig. 1*A*, quantification in Fig. 1*B*). In XZ projections, PMCA labeling was detected along the lateral surfaces of polarized epithelial cells, and this labeling was increased in PLEKHA7-KO cells (Fig. 1*C*, bottom panels). PMCA labeling was next analyzed in separate WT and PDZD11-KO cell cultures. Endogenous PMCA labeling was significantly stronger in PDZD11-KO cells (bottom panels Fig. 1*D*, quantification in Fig. 1*E*) compared to WT cells (top panels Fig. 1*D*, quantification in Fig. 1*E*). To determine whether these increases were due to altered protein expression levels of PMCA in PLEKHA7-KO and PDZD11-KO cells compared to WT cells, we performed immunoblot (IB) analysis of PMCA in total cell lysates, using the same PanPMCA antibody. There was no significant difference in PMCA protein levels between WT and either PLEKHA7-KO or PDZD11-KO cells (Fig. 1*F*, quantification in Fig. 1*G*), indicating that the increase in PMCA signal at cell–cell contacts in PLEKHA7-KO and PDZD11-KO mCCD cells is due to either a redistribution or an increased retention/stabilization or trafficking of PMCA to the plasma membrane, rather than an increase in total protein expression levels.

### KO of either PLEKHA7 or PDZD11 results in the ectopic localization of endogenous PMCA at the apical junctional region

**Figure 2:**
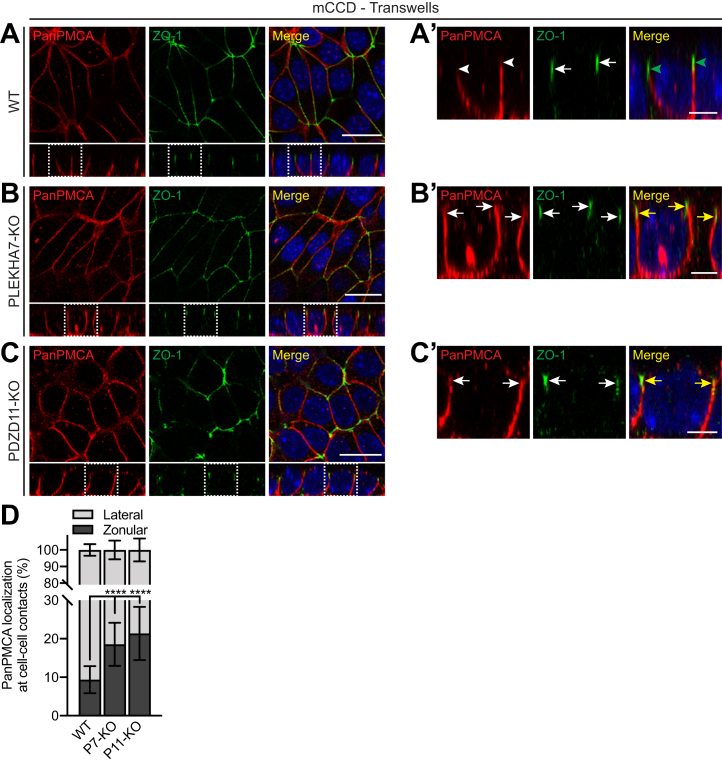
**PMCA localization at lateral cell-cell contacts extends into apical junctions in cells KO for either PLEKHA7 or PDZD11.***A*-*C*, IF microscopy of endogenous PMCA (PanPMCA antibody) in WT (*A*), PLEKHA7-KO (*B*) and PDZD11-KO (*C*) mCCD cells polarized on Transwell filters. *Rectangular* images show the Z section taken at the horizontal middle position of the respective XY view (*square images*). ZO-1 (*green*) is used as junctional marker, and merge images show colocalization between PMCA and ZO-1 labeling. *Dashed white squares* in (*A*–*C*) (XZ sections) correspond to the areas enlarged in (*A*′-*C*’). *Arrows* indicate labeling; *arrowheads* indicate low/undetectable labeling. *Green arrowheads* (*A*′) show the absence and *yellow arrows* (*B*′, *C*′) the presence of colocalization of PMCA with ZO-1. The scale bars represent 20 μm for (*A*–*C*), 5 μm for *A*′-*C*’. *D*, quantification of PanPMCA distribution between lateral and zonular cell–cell contacts in WT, PLEKHA7-KO (P7-KO), and PDZD11-KO (P11-KO) mCCD cells. Values are shown as mean ± SD (n = 22 (WT); 26 (P7-KO); 14 (P11-KO)). One-way ANOVA with post hoc Dunnett’s test (∗∗∗∗*p* < 0.0001). IF, immunofluorescence; mCCD, mouse cortical collecting duct; PMCA, plasma membrane calcium ATPase.

In epithelial cells, PLEKHA7 is detected at circumferential AJs, that is, the *zonula adhaerens*, immediately basal to tight junctions (2). To analyze in more detail the role of PLEKHA7 and PDZD11 in PMCA localization, we cultured mCCD cells on Transwell filters, which allow optimal apicobasal polarization. IF microscopy analysis showed that in WT cells PMCA labeling was excluded from apical junctions, identified by the tight junctions marker ZO-1 (Fig. 2*A*, *arrows* and *arrowheads* in enlarged images in Fig. 2*A*'). In contrast, in PLEKHA7-KO cells, in addition to the increased basolateral signal of PMCA, an ectopic localization at apical junctions, partially colocalized with ZO-1, was detected (Fig. 2*B*, *arrows* in enlarged images in Fig. 2*B*', quantification in Fig. 2*D*). Similarly, in PDZD11-KO polarized mCCD cells, PMCA is detectable at the apical junctions partially colocalized with ZO-1 in addition to its increased basolateral localization (Fig. 2*C*, *arrows* in enlarged images in Fig. 2*C*', quantification in Fig. 2*D*). These results suggest that the PLEKHA7–PDZD11 complex negatively regulates PMCA accumulation at apical junctions.

In the aforementioned experiments we used as antibody an anti-PanPMCA to label PMCA by either IF or IB. Since this antibody recognizes all isoforms, and isoform-specific antibodies were not available, we could not identify either the isoform(s) or the splicing variants whose localization is affected in KO cells. To determine which isoforms of PMCA are expressed in mCCD cells, we performed quantitative RT-PCR analysis to evaluate mRNA expression (Fig. S1). The PMCA1b and PMCA4b isoforms are expressed ubiquitously (27) and are detected in the distal nephron (28), suggesting that they are expressed in mCCD cells. PMCA1 mRNA was the most abundant (lowest ΔC_q_) in mCCD WT cells, followed by PMCA4 mRNA, including both PMCA4a and PMCA4b isoforms at similar levels (Fig. S1*A*). In addition, mRNA expression of the PMCA2 isoform was also detected, while PMCA3 was undetectable (Fig. S1*A*). To evaluate whether the mRNA expression of PMCA was affected by the KO of either PLEKHA7 or PDZD11, we compared their mRNA level between WT, PLEKHA7-KO, and PDZD11-KO mCCD cells. We observed no significant difference for either PMCA1 (Fig. S1*B*), or PMCA2 (Fig. S1*C*), or PanPMCA4 (Fig. S1*D*), or PMCA4a (Fig. S1*E*), or PMCA4b (Fig. S1*F*). Together, these results indicate that KO of either PLEKHA7 or PDZD11 increases PMCA localization at lateral cell–cell contacts and zonular junctions, without significantly affecting either the mRNA expression levels of any isoform or the total PMCA protein expression.

### The exogenously expressed PMCA4x/b isoform is ectopically localized at the apical plasma membrane in cell KO for either PLEKHA7 or PDZD11 and when the PDZ-binding motif is deleted

**Figure 3:**
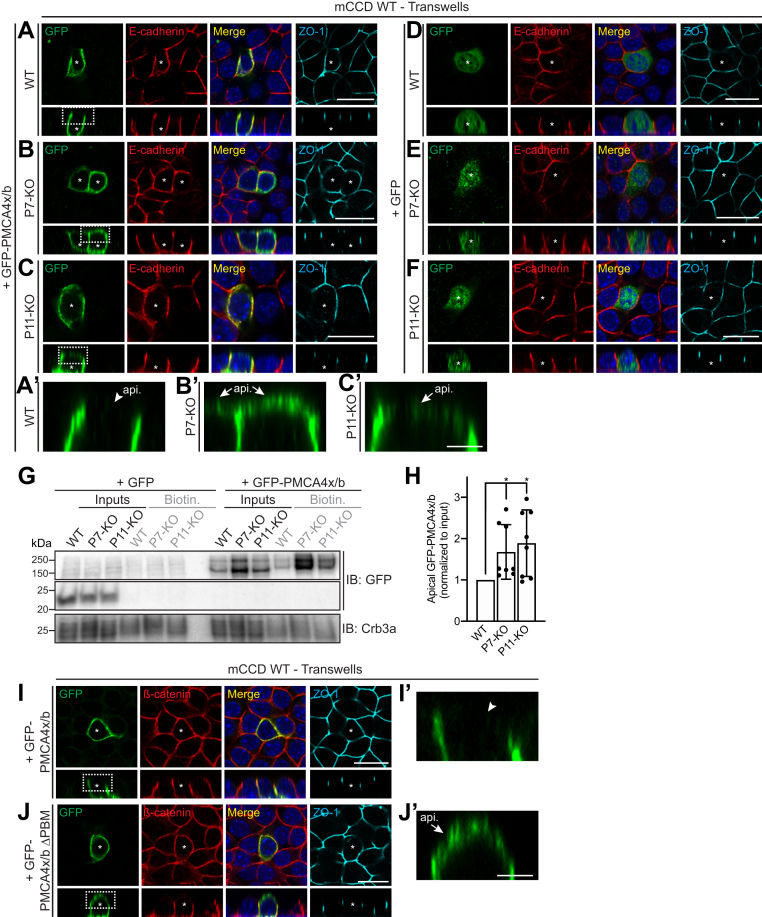
**PMCA4x/b ectopically localizes at the apical plasma membrane in cells KO for PLEKHA7 or PDZD11.***A*–*F*, IF microscopy analysis of exogenous GFP-PMCA4x/b (*A*–*C*, *A*′–*C*′) or GFP alone (control, *D*–*F*) expressed in polarized mCCD cells (grown on Transwell filters), either WT (*A*, *D*) or PLEKHA7-KO (P7-KO) (*B*, *E*) or PDZD11-KO (P11-KO) (*C*, *F*). *Rectangular* panels below XY images show the Z section taken at the horizontal middle position of the respective XY (*square*) images. *Asterisks* indicate transfected cells. *Arrows* indicate apical labeling; *arrowheads* indicate undetectable labeling. ZO-1 and E-cadherin are used as markers for apical junctions and lateral contacts, respectively. *Merge* images show colocalization between E-cadherin and GFP labeling. *Dashed white rectangles* in (*A*–*C*) correspond to the areas enlarged in (*A*′–*C*’). *Arrows* indicate apical (api.) labeling; *arrowheads* indicate undetectable labeling. The scale bars represent 20 μm for (*A*–*F*), 5 μm for (*A*′–*C*’). *G* and *H*, IB analysis (*G*) and quantification (*H*) of apically biotinylated (Biotin) exogenous GFP-PMCA4x/b (GFP as negative control) in WT, PLEKHA7-KO (P7-KO), and PDZD11-KO (P11-KO) mCCD cells. Crb3a was used as positive control for apical labeling. Quantification of apical GFP-PMCA4x/b (*H*) indicates for each genotype the biotinylated GFP(-PMCA4x/b) IB signal normalized to the respective input, relative to WT cells. *Dots* show replicates (n = 8) and *bars* represent mean ± SD. One-way ANOVA with post hoc Dunnett’s test (∗*p* < 0.05). *I* and *J*; *I*′ and *J*′, IF microscopy analysis of exogenous GFP-PMCA4x/b (*I*) or GFP-PMCA4x/b lacking the PDZ-binding motif (GFP-PMCA4x/b ΔPBM) (*J*) expressed in polarized mCCD WT cells (grown on Transwell filters). *Rectangular* panels show the Z section taken at the horizontal middle position of the respective XY view (*square images*). *Asterisks* indicate transfected cells. *Arrows* indicate apical labeling; *arrowheads* indicate undetectable labeling. ZO-1 and ß-catenin are used as markers for apical junctions and lateral contacts, respectively. *Merge* images show colocalization between ß-catenin and GFP labeling. *Dashed white rectangles* in (*I* and *J*) correspond to the areas enlarged in (*I*′ and *J*’). *Arrows* indicate apical (api.) labeling; *arrowheads* indicate undetectable labeling. The scale bars represent 20 μm, 5 μm for (*I*′ and *J*’). IF, immunofluorescence; mCCD, mouse cortical collecting duct; PBM, PDZ-binding motif; PMCA, plasma membrane calcium ATPase.

Since PDZD11 was reported to interact only with b-isoforms of PMCA (26) and the PMCA4 isoform has been implicated in the regulation of blood pressure (22, 23), we asked whether PLEKHA7 and PDZD11 regulate the localization of exogenously expressed PMCA4x/b. In WT mCCD cells polarized on Transwells, GFP-tagged PMCA4x/b was detected at the basolateral membrane, labeled by E-cadherin, and no apical labeling was observed (Fig. 3*A*, arrowhead in enlarged image in Fig. 3*A*’ indicating the apical membrane). The lateral localization of exogenous PMCA4x/b was similar to the localization of endogenous PMCAs (Figs. 1 and 2). However, in PLEKHA7-KO cells, GFP-PMCA4x/b was detected not only along the basolateral membrane but also at the apical surface (Fig. 3*B*, arrows in enlarged image in Fig. 3*B*’). Similarly, in PDZD11-KO cells, an apical staining of GFP-PMCA4x/b was detected, in addition to basolateral localization (Fig. 3*C*, arrow in enlarged image in Fig. 3*C*’). Exogenous GFP, used as a negative control, was localized in the cytoplasm of WT and KO cells (Fig. 3, *D*–*F*).

To confirm biochemically the IF microscopy results, we used a biotinylation assay to detect the apical localization of exogenous PMCA4x/b in KO cells. Either GFP-tagged PMCA4x/b or GFP alone (negative control) were exogenously expressed in WT, PLEKHA7-KO, or PDZD11-KO cells, and apical surface proteins were biotinylated and isolated by affinity chromatography on streptavidin-coated beads. Lysates were analyzed by IB using either anti-GFP antibody, to detected exogenous PMCA4x/b, or anti-Crb3a antibody as positive control for apical surface protein (Fig. 3*G*). In PLEKHA7-KO and PDZD11-KO cells, the level of apical GFP-tagged PMCA4x/b, when normalized to the input level, was significantly higher than in WT cells (Fig. 3*G*, quantification in Fig. 3*H*). The negative control GFP alone was not detected in apically biotinylated lysates from all cell types, while the positive control Crb3a was similarly detected in lysates of all cells (Fig. 3*G*).

Finally, to explore the importance of the PDZ-dependent interaction of PDZD11 with PMCA4x/b, we examined the localization of exogenously expressed PMCA4x/b lacking the last six amino acids, which comprise the PBM (26), in WT mCCD cells. While PMCA4x/b was localized along the basolateral membrane with no apical labeling (Fig. 3*I*, arrowhead in Fig. 3*I*’), the mutant lacking the PBM (ΔPBM) was detected not only along the basolateral membrane but also at the apical surface (Fig. 3*J*, arrow in enlarged image in Fig. 3*J*’).

In summary, these observations indicate that the integrity of the PLEKHA7–PDZD11 complex, and the PBM of PMCA4x/b are required to downregulate the lateral plasma membrane accumulation and prevent the ectopic redistribution of PMCA4x/b to the apical surface of polarized epithelial cells.

### *In vitro* PMCA4x/b binding to the N-terminal WW-PH region of PLEKHA7 is negatively regulated by PDZD11

**Figure 4:**
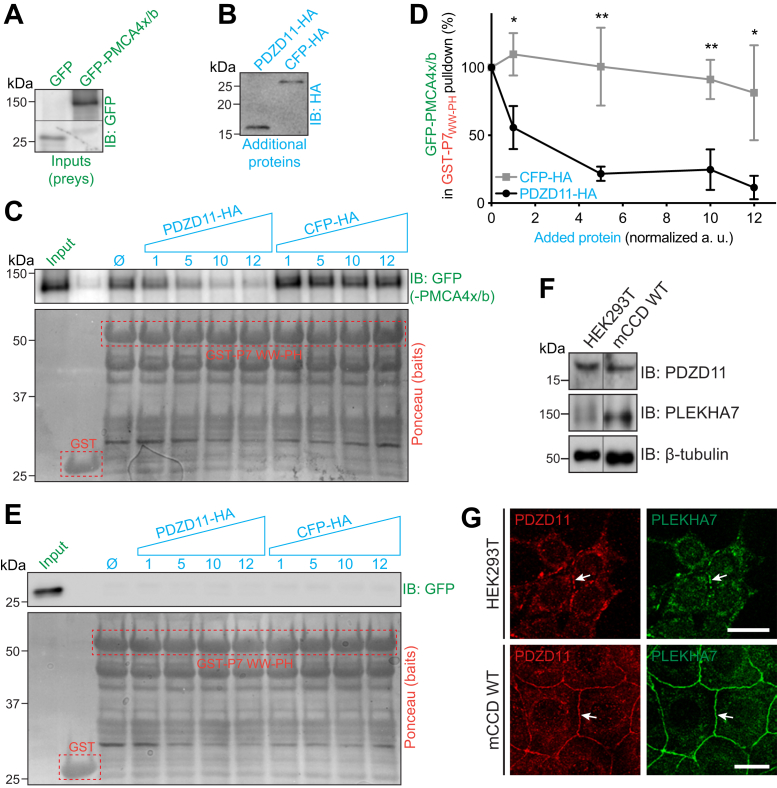
**PDZD11 decreases the binding of PMCA4x/b to the N-terminal WW-PH region of PLEKHA7.** (*A*) and (*B*) Normalized preys (*A*, *green*) and additional proteins (*B*, *blue*) used for GST pull downs. Preys normalized by IB with anti-GFP antibodies (*A*) were either GFP or GFP-tagged PMCA4x/b. Additional proteins normalized with anti-HA antibodies (*B*) were either PDZD11-HA or CFP-HA (negative control). *C* and *E*, IB analysis with anti-GFP antibodies of GST pull downs using GST or GST-PLEKHA7 WW-PH (amino acids 1–284, GST-P7 WW-PH) as baits (Ponceau S staining below IB show baits, *red dotted boxes*) and GFP-PMCA4x/b (*C*) or GFP (negative control, *E*) (preys, *green*), carried out in the absence (ø) or with an increasing amount (normalized relative amount, 1–12) of a HA-tagged third additional protein (*blue*). *D*, quantification of GFP-PMCA4x/b chemiluminescence signal intensity in GST-PLEKHA7_ww-PH_ pull downs (*C*) in the presence of PDZD11-HA (*black line*) or CFP-HA (*gray line*), normalized to the respective bait signal, relative to the value in the absence of additional protein. Bars represent mean ± SD (n = 6). Repeated measures (RM) one-way ANOVA followed by Sidak’s multiple pairwise comparisons test (∗*p* < 0.05, ∗∗*p* < 0.01). *F*–*G*, IB (*F*) and IF (*G*) analysis of PDZD11 and PLEKHA7 expression in HEK293T cells compared to mCCD cells. In F, ß-tubulin is used as IB loading control. In (*G*), arrows indicate cell–cell contact labeling. The scale bars represent 20 μm (*G*). HA, hemagglutinin; IB, immunoblot; IF, immunofluorescence; mCCD, mouse cortical collecting duct; PH, pleckstrin homology; PMCA, plasma membrane calcium ATPase.

To explore the biochemical interactions underlying the regulation of PMCA4x/b by PLEKHA7 and PDZD11, we investigated by glutathione-*S*-transferase (GST) pull-down assays *in vitro* whether the N-terminal region of PLEKHA7, comprising tandem WW and PH domains, interacts with PMCA4x/b *in vitro* and how PDZD11 regulates this interaction. The prey protein (GFP-tagged PMCA4x/b) was expressed in HEK293T cells (Fig. 4*A*) and incubated with baits, that is, either GST-fused to the WW-PH region of PLEKHA7 or GST alone (negative control), expressed in bacteria and purified by affinity chromatography (Fig. 4, *C* and *E*, baits are bands in *red dotted boxes* in bottom panels). IB analysis showed that the N terminus of PLEKHA7 interacts with GFP-PMCA4x/b (Fig. 4*C*, ø) but not with GFP alone (Fig. 4*E*, ø). Since endogenous PDZD11 is expressed at significant levels in HEK293T cells (Fig. 4, *F* and *G*), the interaction between the bait and prey is likely to be indirect, mediated by the PDZD11 protein present in the HEK lysate. To test the impact of exogenously added PDZD11 in this interaction, we used a trimolecular GST pull-down assay, where hemagglutinin (HA)-tagged constructs of either PDZD11 or CFP (negative control) were overexpressed in HEK293T cells and added to the GST pull-down as additional (third) proteins (Fig. 4*B*). IB analysis showed that adding increasing amounts of exogenous PDZD11 negatively regulated the interaction of PMCA4x/b with the WW-PH region of PLEKHA7, while adding CFP had no impact (Fig. 4*C*, quantification in Fig. 4*D*). Since PDZD11 binds with high affinity to the WW domains of PLEKHA7 (3), these results suggest that excess free PDZD11 can compete PLEKHA7 away from the PMCA4x/b-PDZD11–PLEKHA7 complex.

### The overexpression of PDZD11 inhibits the peripheral plasma membrane localization of coexpressed PMCA4x/b in HeLa cells

**Figure 5:**
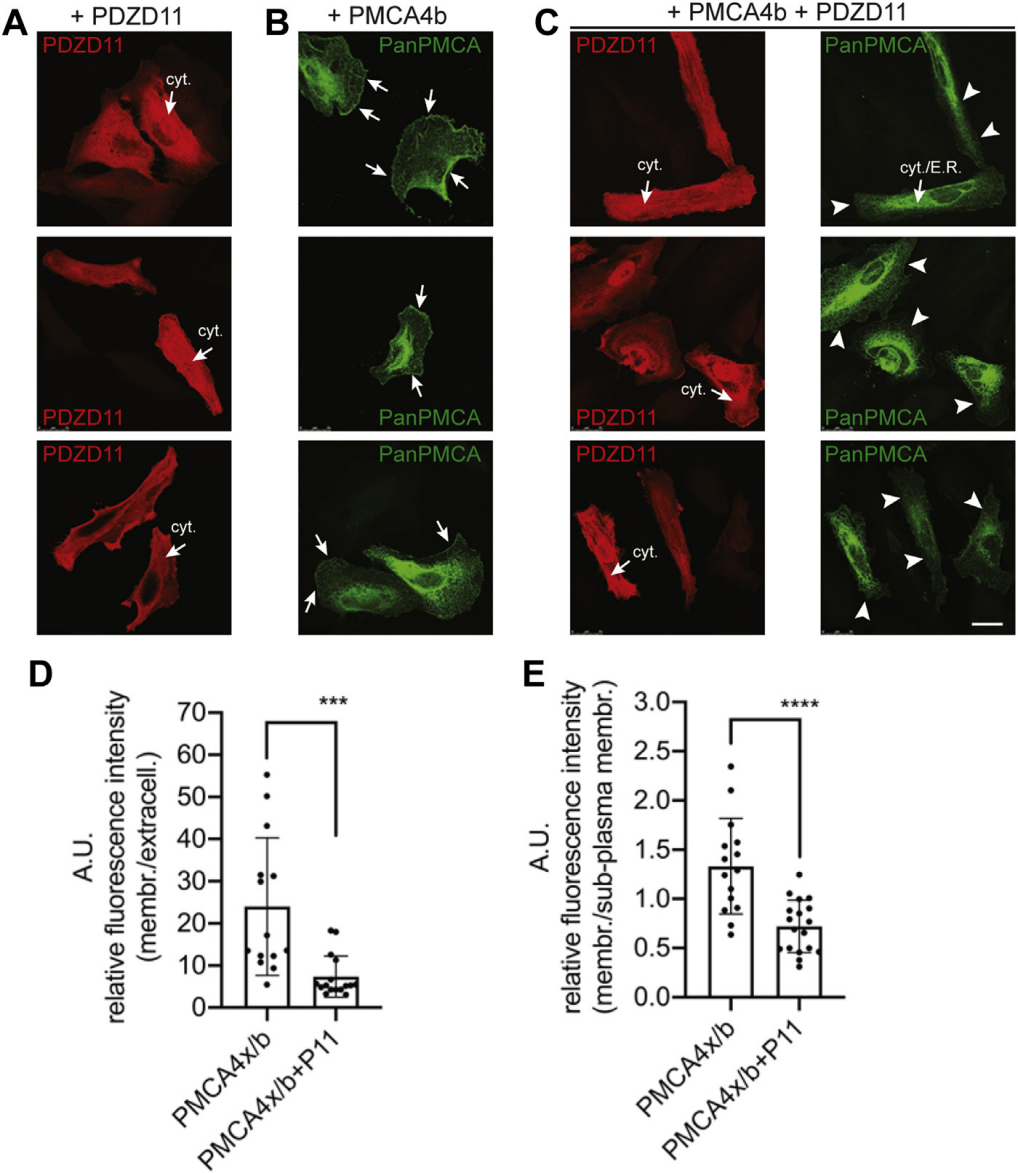
**PDZD11 overexpression inhibits the localization of exogenous PMCA4x/b at the plasma membrane of HeLa cells.***A*–*C*, IF microscopy analysis of exogenous PDZD11 (*A*), exogenous PMCA4x/b (*B*), and exogenous PDZD11 + PMCA4x/b (*C*) after transfection of HeLa cells (grown on coverslips). Three representative images per experimental condition are presented. *Arrows* (*A* and *C*, *red channel*) indicate cytoplasmic (cyt.) and fibrillar labeling for PDZD11. *Arrows* (*B*, *green channel*) indicate accumulation of PMCA4x/b at the peripheral plasma membrane in cells expressing only PMCA4x/b, and *arrowheads* (*C*, *green channel*) indicate lack of peripheral plasma membrane labeling for PMCA4x/b in cells expressing both PDZD11 and PMCA4x/b. Cytoplasmic/endoplasmic reticulum labeling for PMCA4x/b is detected both in the absence and in the presence of PDZD11 (cyt./E.R., *C*). The scale bars represent 25 μm. *D* and *E*, shows quantification of plasma membrane labeling for PMCA in cells overexpressing PMCA4x/b or PMCA4x/b and P11, ratioed to an extracellular region (*D*) and a subplasma membrane region (*E*). *Dots* show analyzed ROI (n > 13) from at least eight cells derived from three independent transfections. *Bars* represent mean ± SD. Ratio unpaired Student’s *t* test (∗∗∗*p* < 0.001, ∗∗∗∗*p* < 0.0001). IF, immunofluorescence; PMCA, plasma membrane calcium ATPase.

To corroborate the finding that PDZD11 has a role in the control of PMCA4x/b distribution at the plasma membrane, we expressed PDZD11 and PMCA4x/b in HeLa cells, either alone or together. IF microscopy analysis of HeLa cells overexpressing either PDZD11 or PMCA4x/b, alone or in combination, revealed that PDZD11 has a diffuse and fibrillar cytosolic distribution (arrows, cyt. in Fig. 5*A*), consistent with its association with cytoplasmic microtubules (7). Instead, PMCA4x/b is clearly detectable at the peripheral plasma membrane (arrows, Fig. 5*B*) and is also accumulated in the cytoplasm/endoplasmic reticulum (ER) compartment, due to its overexpression (29). Strikingly, upon coexpression of PMCA4x/b and PDZD11, PMCA4x/b labeling at the plasma membrane was strongly downregulated (arrowheads, Fig. 5*C*, right panels). Quantification of peripheral *versus* either sub–plasma membrane or extracellular labeling confirms the significant loss of peripheral PMCA4x/b accumulation in HeLa cells coexpressing PMCA4x/b and PDZ11(Fig. 5, *D* and *E*; median ± SD, membrane/extracellular: PMCA4x/b 23.98 ± 16,27, n = 8 cells; PMCA4x/b+P11 7.37 ± 4.93, n = 11 cells; membrane/sub–plasma membrane PMCA4x/b 1.33 ± 0.49, n = 8 cells; PMCA4x/b+P11 0.72 ± 0.27, n = 11 cells). This observation suggests that PDZD11 overexpression is sufficient to perturb the correct delivery of PMCA4x/b to the plasma membrane and promote its cytoplasmic/ER retention.

### PDZD11 increases the amplitude of the calcium transient with PMCA4x/b but not with PMCA4x/a, which lacks the PBM

**Figure 6:**
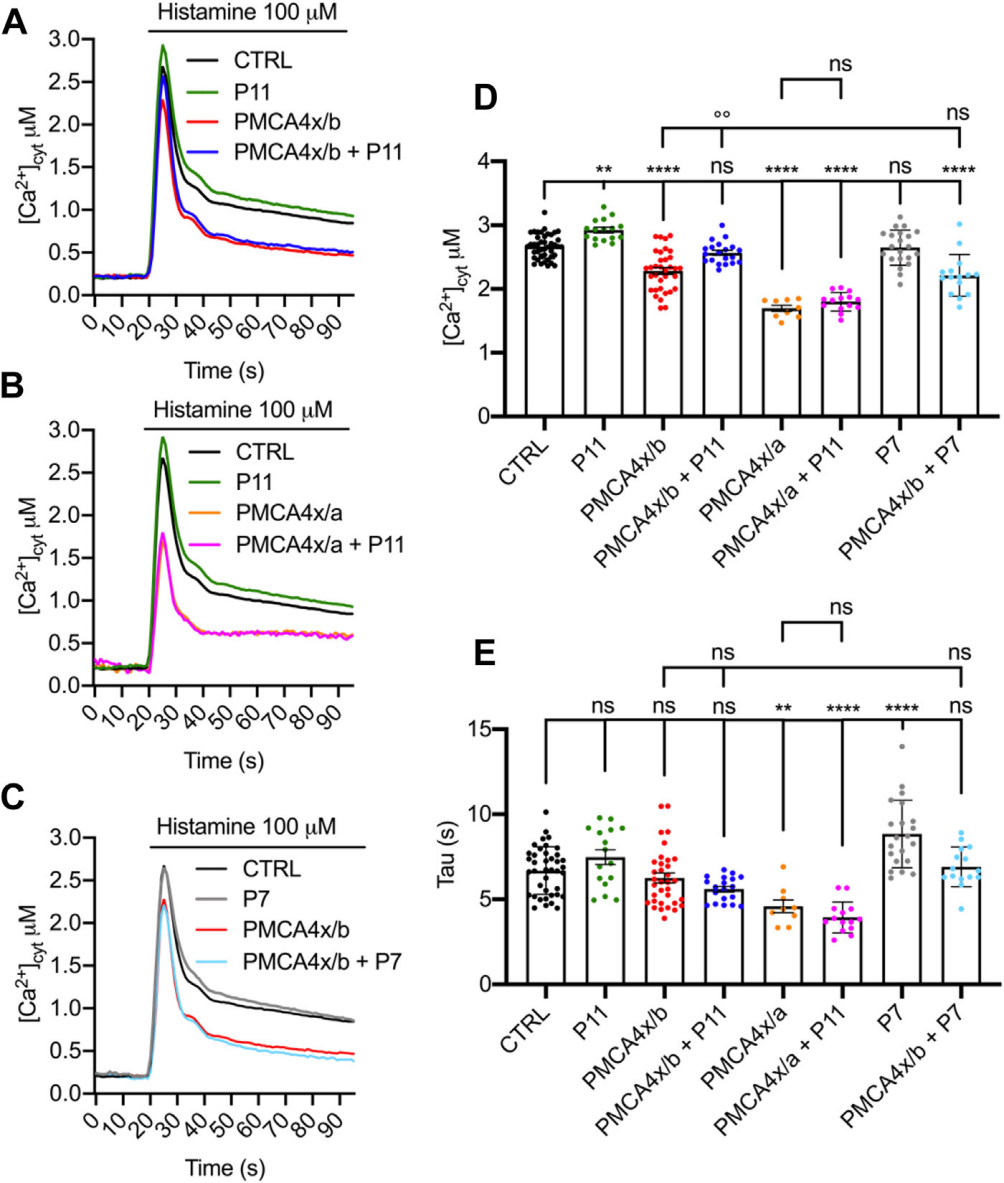
**Overexpression of PDZD11 reduces the amplitude of the calcium transient in HeLa cells.** Expression of either cytosolic aequorin alone (CTRL, *black*) or with PDZD11 alone (P11, *green*), or with PMCA4x/b alone (PMCA4x/b, *red*) or with PDZD11 and PMCA4x/b together (PMCA4x/b + P11, *blue*) or PMCA4x/a alone (PMCA4x/a, *orange*) or PDZD11 and PMCA4x/a together (PMCA4x/a + P11, *violet*) or PLEKHA7 alone (P7, *gray*) on PLEKHA7 and PMCA4x/b together (PMCA4x/b + P7, *light blue*). Cytosolic calcium concentration ([Ca^2+^]_cyt_) measurements (*A*–*C*), mean peak values (*D*), and mean of the time constant τ of the fitted one-phase exponential decay used to estimate PMCA-mediated calcium extrusion rate (*E*) in HeLa cells transiently expressing the indicated constructs. Cells were stimulated with 100 μM histamine at the indicated time to induce increase of cytoplasmic [Ca^2+^] ([Ca^2+^]_cyt_). Traces are the average of at least nine replicates, resulting from three independent transfections which gave similar results. The error bars are omitted from the traces (*A*–*C*) for the sake of graphic clarity, and are shown in the histograms (*D* and *E*), where *dots* indicate the replicates and *bars* represent mean ± SD. Statistical significance was determined by one-way ANOVA and multiple comparisons were performed with Tukey’s test. Only relevant *p* values are indicated in the text, full data are available in Table S2; ∗∗*p* < 0.01 referred to CTRL, ∗∗∗*p* < 0.001 referred to CTRL, ∗∗∗∗*p* < 0.0001 referred to CTRL, °°*p* < 0.01 referred to PMCA4x/b, ns=not significant. PMCA, plasma membrane calcium ATPase.

Next, we explored the role of the PLEKHA7–PDZD11 complex in Ca^2+^ handling. We first used the overexpression system in HeLa cells, where PMCA4 isoforms were overexpressed together with the Ca^2+^ sensitive photoprotein aequorin (AEQ), which is targeted to the cytosolic compartment (cytAEQ) (29, 30, 31), either alone or together with either PLEKHA7 or PDZD11. After transfection, HeLa cells were stimulated with the inositol(1,4,5) trisphosphate (InsP_3_)-linked agonist histamine to induce Ca^2+^ release from the ER and subsequent Ca^2+^ influx from the extracellular space, generating a cytosolic Ca^2+^ transient (Fig. 6, *A*–*C*). Quantification of the mean peak values of the traces (Fig. 6*D*) showed that PMCA4x/b alone reduced the amplitude of the Ca^2+^ transients (compare *black* to *red* trace in Fig. 6*A*, and in panel D peak Ca^2+^ values (μM) mean ± SD: 2.67 ± 0.19, n= 42, for control *versus* 2.28 ± 0.32, n= 34 for PMCA4x/b overexpressing cells, *p* < 0.0001, additional statistical comparisons in Table S2). Importantly, coexpression of PMCA4x/b together with PDZD11 resulted in a statistically significant increase in the amplitude of the calcium transient when compared to PMCA4x/b alone (Fig. 6*A*, compare *blue* trace to *red* trace, Fig. 6*D* peak values (μM) mean ± SD: 2.57 ± 0.17, n= 20 for coexpression *versus* 2.28 ± 0.32, n = 34 for PMCA4x/b alone, *p* = 0.001, additional statistical comparisons in Table S2). Interestingly, overexpression of PDZD11 alone also increased the amplitude of Ca^2+^ transients (Fig 6, *A* and *D*, compare *green* to *black* trace; peak values (μM) mean ± SD: 2.92 ± 0.17, n = 16 for PDZD11 *versus* 2.67 ± 0.19, n = 42 for control, *p* = 0.0093, additional statistical comparisons in Table S2), suggesting that exogenous PDZD11 may interact with endogenous PMCA pump(s) and inhibit their activity.

To confirm that the effect observed on Ca^2+^ transients was related to the PDZ-dependent interaction of PDZD11 with PMCA4x/b, we performed cytosolic Ca^2+^ measurements in HeLa cells coexpressing PDZD11 and the PMCA4x/a isoform, a PMCA4 variant which lacks the C-terminal PBM. The overexpression of the PMCA4x/a pump increased Ca^2+^ extrusion activity of the cells since the amplitude of the Ca^2+^ transient was strongly reduced (Fig. 6*B*, compare *orange* to *black* trace; Fig. 6*D*, peak values (μM) mean ± SD: 1.69 ± 0.14, n = 9 for PMCA4x/a *versus* 2.67 ± 0.19, n = 42 for control, *p* < 0.0001, additional statistical comparisons in Table S2). Importantly, the coexpression of PMCA4x/a with PDZD11 had no effect on PMCA4x/a activity (Fig. 6*B*, compare *violet* to *orange* trace; Fig. 6*D*, peak values (μM) mean ± SD: 1.69 ± 0.14, n = 9 for cells expressing PMCA4x/a alone *versus* 1.8 ± 0.15, n = 14 for cells expressing PMCA4x/a and PDZD11, additional statistical comparisons in Table S2). Figure 6*E* shows the mean values of the time constant τ (tau, s) of the exponential decay of the cytosolic Ca^2+^ traces. Statistically significant differences were measured among the values calculated for PMCA4x/a *versus* control, (PMCA4x/a 4.52 ± 1.12, CTRL n = 9, 6.69 ± 1.39 n = 40 *p* = 0.0039, additional statistical comparisons in Table S2), PMCA4x/a plus PDZD11 *versus* control (PMCA4x/a + PDZD11 3.94 ± 0.91, n= 14, *p* < 0.0001, respectively), but not PMCA4x/a *versus* PMCA4x/a plus PDZD11. These differences indicate that the PMCA4x/a pump is more potent than PMCA4x/b and that PDZD11 does not affect either the peak or the τ value for PMCA4x/a, while it affects the amplitude of the Ca^2+^ transient for PMCA4x/b (Fig. 6*D*).

We also carried out cytosolic Ca^2+^ measurements using cytAEQ in HeLa cells overexpressing either PLEKHA7 alone or in coexpression with PMCA4x/b (Fig. 6, *C*–*E*). PLEKHA7 alone did not modify cytosolic peak Ca^2+^ transients, (Fig. 6*C*, compare *black* to *gray* traces, Fig. 6*D*, peak values (μM) mean ± SD: 2.67 ± 0.19, n= 42 for control cells *versus* 2.65 ± 0.28, n = 21 for PLEKHA7 overexpressing cells, additional statistical comparisons in Table S2) and was not able to counteract the reduction caused by the overexpression of PMCA4x/b (Fig. 6*D*, 2.21 ± 0.33, n= 15 for PMCA4x/b/PLEKHA7 co-expressing cells *versus* 2.28 ± 0.32, n= 34 for PMCA4x/b overexpressing cells, and Table S3 for additional two-way ANOVA statistical analysis). The values of the time constant τ of the exponential decay of the cytosolic Ca^2+^ traces between control cells and PLEKHA7 overexpressing cells were statistically different (CTRL 6.69 ± 1.39, n = 40 *versus* PLEKHA7 8.84 ± 1.99, n = 21, *p* < 0.0001, additional statistical comparisons in Table S2 and Table S3) (Fig. 6*E*).

In summary, these results indicate that PDZD11 is sufficient to impact on calcium homeostasis and that the PDZ-binding domain of PMCA4, present in the PMCA4x/b but absent in the PMCA4x/a isoform, is required for this impact. Furthermore, the analysis of tau values revealed a possible impact of PLEKHA7 on PMCA pump extrusion ability, albeit under conditions of PMCA4x/b overexpression this impact is not statistically apparent.

### bEnd.3 and mCCD cells KO for PDZD11 show enhanced PMCA-mediated calcium extrusion

To further validate the role of PDZD11 in PMCA-mediated calcium handling, we generated endothelial bEnd.3 cells KO for PDZD11 (Fig. S2) and we carried out cytosolic Ca^2+^ measurements both in these cells and in mCCD cells KO for PDZD11. In this physiological setting, multiple PMCA isoforms are expressed. By quantitative RT-PCR, mRNAs coding for different isoforms of PMCA were detected in bEnd.3 cells, with highest levels for PMCA1, similar to mCCD cells (Figs. S3*A* and S1*A*). KO of PDZD11 was associated with as 30% decrease in PMCA1 mRNA and 80% increase in PMCA2 mRNA (Fig. S3, *B* and *C*) and no significant change in the mRNA levels for PMCA4 isoforms (Fig. S3, *D*–*F*). However, the relative expression and the localization of each PMCA isoform at the protein level could not be determined, due to the lack of isoform-specific antibodies. IF microscopy using an anti-panPMCA antibody showed weak heterogeneous PMCA labeling at the periphery of cultured bEnd.3 cells and no consistent differences between WT and PDZD11-KO cells (Fig. S3, *G*–*H*). Furthermore, the labeling for exogenously expressed PMCA4x/b was mostly diffusely cytoplasmic/cortical, and no consistent difference in localization was detected between WT form of PMCA4x/b and the mutant lacking the PBM (ΔPBM) (Fig. S3, *I*–*J*).

**Figure 7:**
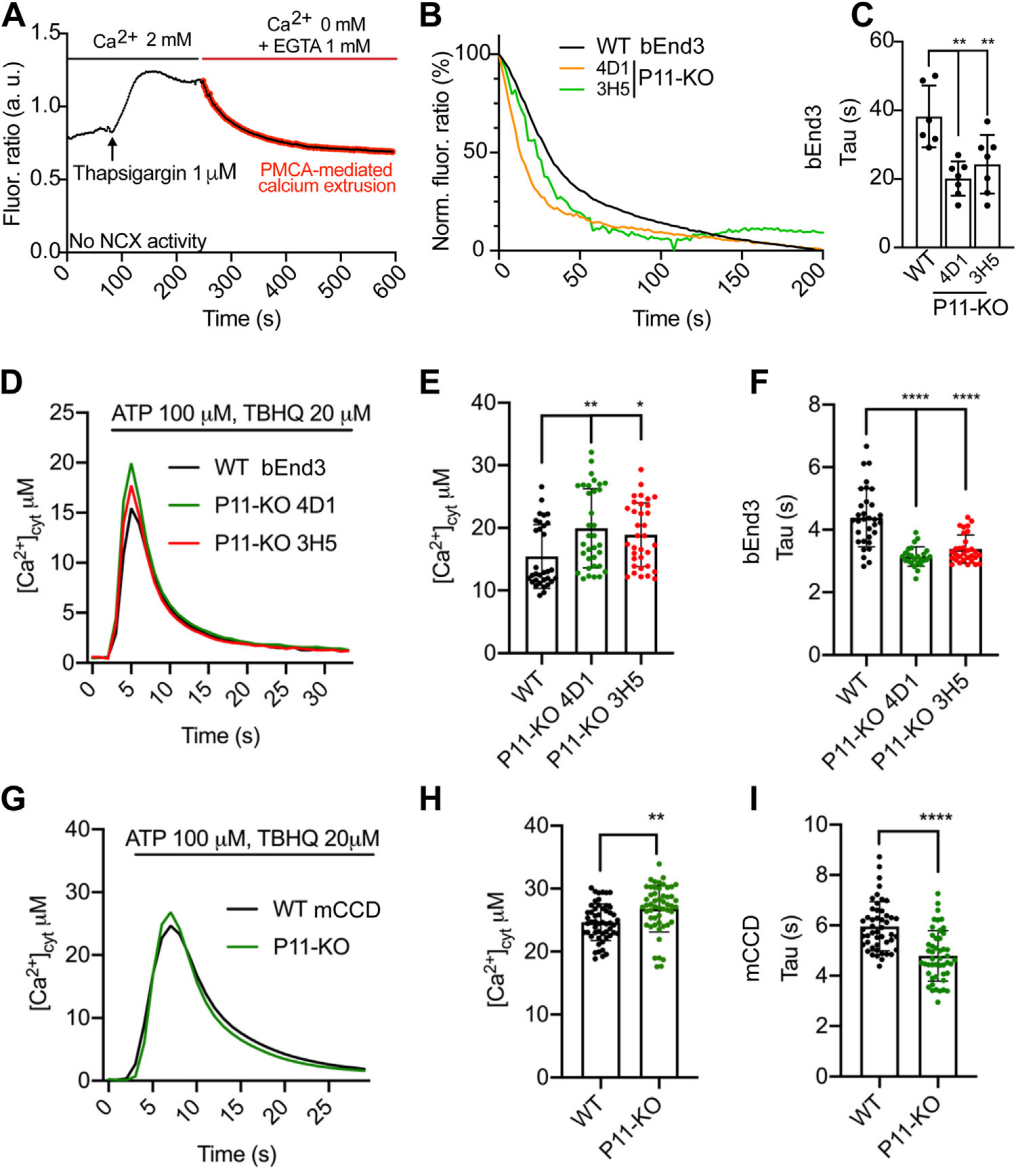
**PMCA-mediated calcium extrusion is enhanced in PDZD11-KO bEnd.3 and mCCD cells.***A*–*C*, measurement of cytosolic calcium transients in either WT or PDZD11-KO bEnd.3 cells by single-cell imaging using Fura-2. *A*, representative trace of Fura-2 fluorescence ratio (arbitrary units, a.u.) in the set-up used for measurement of PMCA-mediated calcium extrusion, carried out in the absence of sodium in the medium (NMDG instead of NaCl to prevent NCX activity) showing the addition of thapsigargin and the switch from calcium-containing medium ([Ca^2+^] 2 mM) to calcium-free medium ([Ca^2+^] 0 mM + EGTA 1 mM) (see Experimental procedures). The part of the trace corresponding to PMCA-mediated calcium extrusion and used to fit a one-phase exponential decay is highlighted in *red*. *B*, normalized fluorescence ratio of the signal decay corresponding to the PMCA-mediated calcium extrusion in either WT or two distinct clones (CD1, 3H5) of PDZD11-KO (P11-KO) bEnd.3 cells. *C*, estimation of PMCA-mediated calcium extrusion rate (Tau) by the time constant τ of the fitted one-phase exponential decay in WT and PDZD11-KO (P11-KO) bEnd.3 cells. *Dots* show replicates (n=6–7) and *bars* represent mean ± SD. One-way ANOVA with post hoc Dunnett’s test (∗∗*p* < 0.01). *D*–*F*, measurement of calcium transients in either WT or PDZD11-KO bEnd.3 cells by cytosolic aequorin. *D*, cytosolic calcium concentration ([Ca^2+^]_cyt_) transients in bEnd.3 cells WT (*black*), PDZD11-KO clone 4D1 (P11-KO 4D1, *green*), PDZD11-KO clone 3H5 (P11-KO 3H5, *red*) infected with cytosolic aequorin. Mean peak values (*E*) and time constant τ (Tau) (*F*) of the fitted one-phase exponential decay used to estimate PMCA-mediated calcium extrusion rate. Cells were stimulated with 100 μM ATP and 20 μM TBHQ at the indicated time to induce an increase of cytoplasmic [Ca^2+^] ([Ca^2+^]_cyt_). Traces are the average of at least 25 replicates, resulting from three independent experiments that gave similar results. The error bars are omitted from the traces (*D*) for the sake of graphic clarity and are shown in the histograms (*E*, *F*), where *dots* show replicates and *bars* represent mean ± SD. One-way ANOVA post hoc with Dunnet’s test (∗*p* < 0.05, ∗∗*p* < 0.01, ∗∗∗∗*p* < 0.0001). *G*–*I*, measurement of calcium transients in either WT or PDZD11-KO mCCD cells by cytosolic aequorin. *G*, cytosolic [Ca^2+^] transients in mCCD cells WT (*black*), PDZD11-KO (P11-KO, *green*), transduced with adenovirus for cytosolic aequorin expression. *H*, mean peak values and (*I*) mean of the time constant τ of the fitted one-phase exponential decay used to estimate PMCA-mediated calcium extrusion rate. Cells were stimulated with 100 μM ATP and 20 μM TBHQ at the indicated time to induce increase of cytoplasmic [Ca^2+^] ([Ca^2+^]_cyt_). Traces are the average of at least 53 replicates, resulting from four independent experiments that gave similar results. The error bars are omitted from the traces (*G*) for the sake of graphic clarity and are shown in the histograms (*H*, *I*), where *dots* show replicates and *bars* represent mean ± SD. Statistical significance was determined by unpaired Student’s *t* test (∗∗∗*p* < 0.001, ∗∗∗∗*p* < 0.0001). N-methyl-D-glucamine; mCCD, mouse cortical collecting duct; PMCA, plasma membrane calcium ATPase; TBHQ, Tert-butylhydroquinone.

To measure calcium handling, we carried out live microscopy analysis of intracellular calcium transients using two protocols, one with the cytosolic ratiometric fluorescent calcium probe Fura-2 and single-cell analysis, which is ideally suited to flat cells such as bEnd.3 and not to mCCD cells (Fig. 7, *A*–*C*), and one with cytAEQ, appropriate for both bEnd.3 and mCCD cells (Fig. 7, *D*–*F*). Using the first (Fura-2) protocol, store-operated calcium entry was activated by the addition of the sarcoendoplasmic reticulum calcium-ATPase (SERCA) inhibitor thapsigargin and, as the ratiometric signal reached a plateau, the external solution containing 2 mM Ca^2+^ was replaced by a calcium-free solution (Fig. 7*A*) (32). Under these experimental conditions, SERCA pump activity was completely abolished by the irreversible SERCA inhibitor thapsigargin, and the calcium extrusion activity of the Na^+^/Ca^2+^ exchanger (NCX) was also prevented by replacing sodium chloride with N-methyl-D-glucamine in solutions. Thus, the measured decrease in cytosolic calcium concentration under these conditions is exclusively due to endogenous PMCAs activity (Fig. 7*A*, *red line*), which can be estimated by the time constant τ (s) of a fitted one-phase exponential decay (32). The signal decay corresponding to PMCA-mediated calcium extrusion was faster in two distinct clones of PDZD11-KO cells compared to WT cells (Fig. 7*B*), as pointed out by lower values of the time constant τ (Fig. 7*C*). These results indicate that PDZD11 negatively regulates the calcium extrusion activity of endogenous PMCA, possibly the PMCA2x/b isoform.

Using the second protocol, we monitored cytosolic Ca^2+^ transients in both bEnd3 and mCCD KO for PDZD11 by expression of cytAEQ *via* adenoviral transduction (Fig. 7, *D*–*F* for bEnd.3 cells and Fig. 7, *G*–*I* for mCCD cells). The calcium transient was induced by the addition of ATP in presence of the SERCA inhibitor Tert-butylhydroquinone. Under these conditions, the SERCA pump is inhibited, and calcium efflux depends mainly on PMCA activity. These experiments were performed in the presence of physiological concentrations of extracellular Ca^2+^, and at variance with those performed in Figure 7*A* cells were maintained in physiological concentrations of extracellular Na^+^ (33, 34, 35). Both the mean peak values (Fig. 7, *E* and *H*) and the time constant τ of a fitted one-phase exponential decay (Fig. 7, *F* and *I*) were calculated. The time constants τ for the two bEnd.3 PDZD11-KO clones were significantly lower than those calculated for control cells (4D1 3.14 ± 0.31, n = 25 vs WT 4.39 ± 0.93, n = 32, *p* < 0.0001, 3H5 3.38 ± 0.45, n = 31 *versus* WT 4.39 ± 0.93, n = 32, *p* < 0.0001) (Fig. 7*F*), indicating a faster Ca^2+^ extrusion activity. Surprisingly, the average values of the calcium transient peaks in PDZD11-KO cells were slightly higher compared to those of WT cells (4D1 19.94 ± 6.32, n = 34 *versus* WT 15.43 ± 5.10, n = 32, *p* = 0.0026, 3H5 18.90 ± 5.07, n = 34 *versus* WT 15.43 ± 5.10, n = 32, *p* = 0.0234) (Fig. 7*E*). This finding may appear contradictory; however, it can be explained by the relief of Ca^2+^ feedback inhibitory action on InsP3 receptors (36), that is, the augmented Ca^2+^ extrusion could be responsible for the local reduction of cytosolic Ca^2+^ values and results in prolonged InsP3 receptor-mediated Ca^2+^ release.

The same analysis was performed in WT and PDZD11-KO mCCD cells. The time constant τ (s) for the PDZD11-KO mCCD clone was significantly lower than that calculated for control WT cells (4.79 ± 1,00, n = 48 *versus* WT 5.96 ± 0.97, n = 48, *p* < 0.0001) (Fig. 7*I*), indicating a faster Ca^2+^ extrusion activity. The average value of the calcium transient peaks in PDZD11-KO mCCD cells was slightly higher compared to those of WT cells (26.82 ± 3.69, n = 53 *versus* WT 24.65 ± 2.91, n = 53, *p* = 0.0011) (Fig. 7*H*), confirming the trend observed in bEnd.3 cells.

In summary, analysis of two different cellular models with a KO for PDZD11 provided a similar result, indicating a faster Ca^2+^ extrusion activity in the absence of PDZD11.

## Discussion

PMCA plays a major role in the termination of calcium signaling processes and can thus impact on downstream events that are crucial for the modulation of cellular functions. Nevertheless, the mechanisms underlying the accumulation and distribution of PMCA at the cell surface and how this correlates with PMCA activity is not well understood. Specifically, PDZD11 was previously identified as a PMCA interactor, but its role in PMCA localization and activity was unclear. Here, we used cultured cells with either KO or overexpression of PDZD11 and its partner PLEKHA7, IF microscopy, biotinylation and pull-down analyses, and calcium transient analyses to investigate the impact of the PLEKHA7–PDZD11 complex on the localization and activity of PMCA. We show that (1) in kidney collecting duct (mCCD) epithelial cells, PDZD11 and its interacting partner PLEKHA7 regulate the lateral accumulation and the apical *versus* lateral localizations of PMCA, without altering its protein and mRNA expression; (2) the localization of exogenous PMCA4x/b is regulated by PDZ-binding sequences; (3) the PDZD11-binding region of PLEKHA7, PDZD11, and PMCA can form a trimolecular complex *in vitro*; (4) PDZD11 inhibits the plasma membrane localization of PMCA4x/b in a reconstituted overexpression system in HeLa cells; (5) in the same system, coexpression of PDZD11 with PMCA4x/b but not with PMCA4x/a alters calcium handling; (6) PDZD11 regulates endogenous PMCA-mediated calcium extrusion in bEnd.3 and mCCD cells.

Our observation that KO of either PLEKHA7 or PDZD11 affects the localization of both endogenous PMCA(s) and exogenous PMCA4x/b is consistent with the notion that PDZ-domain proteins are involved in the trafficking, targeting, retention, and anchoring of membrane receptors and ion channels at the plasma membrane (37, 38). Indeed, PDZD11, in complexes with WW-PLEKHAs (PLEKHA5, PLEKHA6, PLEKHA7), regulates the trafficking of the Menkes copper ATPase ATP7A and its accumulation at cell periphery (7). The increase in the lateral labeling for PMCA that we observed in mCCD KO for either PDZD11 or PLEKHA7 was not correlated with upregulation of either the protein or mRNA expression levels of PMCA, which instead occurs in regulation by calcineurin and calcium (39, 40, 41). This suggests that the PLEKHA7–PDZD11 complex has an inhibitory effect on the targeted delivery and/or accumulation of PMCA at the plasma membrane. In agreement with this idea, overexpression of PDZD11 in HeLa cells resulted in decreased plasma membrane accumulation of exogenous PMCA4x/b. Using exogenous expression of PMCA4x/b, we also showed that the KO of either PDZD11 or PLEKHA7 results in an ectopic apical localization of PMCA4x/b, which is dependent on its PDZ-binding domain. Collectively, our observations support the idea that the PLEKHA7–PDZD11 complex interacts with PMCA to regulate its localization.

The mechanism through which the PLEKHA7–PDZD11 complex modulates the basolateral plasma membrane accumulation of PMCA in epithelial mCCD cells remains to be clarified. One possibility is that PDZD11 competitively inhibits the interaction of the PBM of PMCA b-isoforms with other PDZ protein scaffolds that stabilize these isoforms at the membrane. In fact, PMCA b-isoforms interact with PDZ-containing members of the membrane-associated guanylate kinase (MAGUK) family, such as PSD-95/SAP90, SAP97/hDlg, SAP102, nNOS, PSD-93/chapsyn-110, and the calcium/calmodulin-dependent serine protein kinase CASK (42, 43, 44, 45, 46). The surface expression of PMCA4b is increased by PSD-95 without a change in its overall protein level in the cells (46). PMCA1 and PMCA2 b-isoforms, but not PMCA4, interact with the Na^+^/H^+^ exchanger regulatory factor (NHERF2), which enhances their surface concentration and membrane retention by tethering the pump to the underlying actin cytoskeleton (47, 48, 49, 50). The PBM of PMCA b-isoforms can mediate PMCA dimerization, leading to increased activity (51, 52) and facilitate the recruitment of scaffolding and signaling complexes, suggesting specialized signaling roles for different PMCA isoforms (17, 19). For example, subcompartmentalized Ca^2+^ microdomains generated by PMCA isoforms represent an ideal microenvironment for the regulation of their downstream signaling, as shown for nitric oxide synthase-1 (NOS-1) (53, 54). A second mechanism could be the regulation, by the PDZD11–PLEKHA7 complex, of the trafficking and endocytic recycling of PMCA. The C-terminal tail of PMCA contains dileucine motifs, which promote the endocytic internalization of PMCA (55, 56). Masking of these motifs through interactions with additional partners prevents the entry of the transmembrane molecule into the endocytic pathway and maintains it at the plasma membrane, as in the case of p120-catenin, which prevents the internalization of cadherin (57). Importantly, dynamin-dependent endocytosis mediated by the dileucine motifs is involved in the trafficking of some PMCA isoforms (55). Thus, the binding of PDZD11 may alter the structural properties of the cytosolic C-terminal tail of PMCA and promote the exposure of the dileucine motifs that induce the endocytic recycling of the pump.

KO of PLEKHA7 also increased the accumulation of PMCA at cell–cell contacts similarly to KO of PDZD11. PLEKHA7 interacts with PDZD11 and stabilizes it at apical AJs (3), and KO of PLEKHA7 results in decreased protein levels for PDZD11 (6). Since PLEKHA7 is mostly accumulated at junctions and not detectably colocalized with PMCA along the lateral membrane, it is unlikely that PLEKHA7 plays any direct role in binding to and stabilization of PMCA at the plasma membrane, as it was shown for nectins and ADAM10 (3, 4). However, PLEKHA7 could indirectly affect PMCA either by controlling the stability and protein levels of PDZD11 or by affecting the trafficking of the PMCA–PDZD11 complex to the plasma membrane, potentially involving a competition between PMCA “b” isoforms and PDZD11 for binding to the N-terminal region of PLEKHA7, that we showed can occur *in vitro*.

We also observed an ectopic localization of exogenous PMCA4x/b to the apical membrane and a more apical distribution of lateral endogenous PMCA in mCCD cells KO for either PLEKHA7 or PDZD11, suggesting that the PLEKHA7–PDZD11 complex prevents such ectopic localization. We speculate that such a phenotype was not detected using the anti-PanPMCA antibody because of the low levels of endogenous PMCA “b” isoforms, compared to overexpressed protein, and/or low affinity of binding of the antibody to isoforms regulated by the PLEKHA7–PDZD11 complex. Previous studies addressed the mechanisms underlying apical *versus* basolateral localization of PMCA. The dileucine motif in the b-tail splice variants promotes the basolateral sorting of b-isoforms (56). However, neither the deletion of the PDZ-binding domain of PMCA4b nor the mutation of the dileucine motifs in the cytosolic tail result in apical distribution of PMCA in Madin–Darby canine kidney cells, whereas apical membrane targeting depends on alternative splicing of the first cytosolic loop (55, 58). Our results therefore suggest that the PLEKHA7–PDZD11 complex may play a role in the apical exclusion of PMCA4x/b, through an effect either on trafficking or on the conformation of its cytoplasmic tail. In addition, the PLEKHA7–PDZD11 complex may act by limiting the membrane diffusion of PMCA4x/b molecules by maintaining a lipid environment that excludes PMCA from apical junctions. How apical junctions establish and maintain a restricted distribution of membrane lipids is not clear (59), but the PH domain of PLEKHA7 interacts with membrane phosphoinositides (6, 13, 60), suggesting that it may contribute to maintaining lipid rafts. Indeed, PMCA is sensitive to the lipid composition of the surrounding membrane, and the calmodulin-binding domain of PMCA interacts with acidic phospholipids (61, 62, 63, 64, 65, 66). Thus, PLEKHA7 may control PMCA accumulation along the lateral *versus* apical plasma membrane by impacting on the distribution of phospholipids either in the plasma membrane or in endocytic recycling vesicles. Future studies should address these hypotheses. In addition, isoform-specific antibodies will allow to identify precisely which endogenous PMCA isoforms are regulated by PDZD11.

Analysis of cytosolic calcium transients either in HeLa cells that overexpress PMCAs along with either PDZD11 or PLEKHA7, or in endothelial (bEnd.3) and epithelial (mCCD) cells KO for PDZD11, indicate a role of the PLEKHA7–PDZD11 complex in calcium handling. The overexpression approach in HeLa cells allows to explore pathways for active Ca^2+^ transport in a cellular setting, where all physiological regulators and dynamic interactions are preserved. Using this approach, we previously characterized PMCA isoforms specificity, showing for example that the neuron-specific PMCA2 and PMCA3 isoforms are more active than the ubiquitously expressed isoforms 1 and 4 to reduce the height of the Ca^2+^ transients and that PMCA4a is as effective as PMCA4b, even if its affinity for calmodulin is lower, indicating that the availability of calmodulin may not be critical for the modulation of PMCA pumps (31). Using this approach, we also showed that 14-3-3- epsilon inhibits the activity of PMCA4 but not that of PMCA2 by interacting with its N-terminal domain (67). Finally, we showed that a PMCA2 mutation associated with a deafness phenotype is activated only mildly when exposed to a Ca^2+^ pulse, and in this case, the effective Ca^2+^ extrusion is much more evident on the decay phase rather than on the peak amplitude (68). Other studies used a similar system *in vivo* to investigate how PMCAs contribute to peak amplitude, decay kinetics, and oscillatory pattern of Ca^2+^ transients (69). Here, we show that PDZD11 impacts on the amplitude of the Ca^2+^ peak both alone and when coexpressed with PMCA4x/b but not PMCA4x/a. The lack of a detectable effect of PDZD11 on the extrusion rate (tau) in HeLa cells could be due to the fact that PMCA4 isoforms impact peak amplitude more than the long extrusion phase (31). The reduced PMCA accumulation at the plasma membrane in HeLa cells that overexpress PDZD11 suggests that PDZD11 acts by modulating the ER–plasma membrane traffic and plasma membrane accumulation of PMCA. The monitoring of cytosolic Ca^2+^ handling in in two different KO cell model lines, that is, endothelial (bEnd.3) and epithelial (mCDD) cells confirmed this view. In both cell types, the KO of PDZD11 (*e.g.*, the opposite of overexpression) increased the Ca^2+^ extrusion ability of the cell as indicated by a reduction of the time constant τ, monitored using two distinct protocols. In mCCD cells, which are more suited to analyze localization, this altered calcium handling correlated with increased lateral accumulation of endogenous PMCA and apical mistargeting of exogenous PMCA4x/b.

## Conclusions

In summary, we show by KO and overexpression experiments in epithelial, endothelial, and HeLa cells that the integrity of the PLEKHA7–PDZD11 complex is required for the correct trafficking and plasma membrane localization of PMCA, correlating with altered amplitude and decay of calcium transients. This suggests that the PLEKHA7–PDZD11 complex fine-tunes calcium homeostasis by regulating the accumulation of PDZ-binding PMCAs at specific membrane domains. This, in turn, could have functional consequences on downstream PMCA partners and signaling events involved in the control of blood pressure.

## Experimental procedures

Key resources used in this article are listed in Table S1.

### Cell culture and transfection

Culture conditions for mCCD cells (mCCD Tet-ON), mouse brain microvascular endothelial (endothelioma) cell line (bEnd.3), and human embryonic kidney epithelial cells (HEK293T) were described previously (5, 70). PLEKHA7-KO and PDZD11-KO mCCD were obtained by CRISPR/Cas9 genomic editing (3, 9). PDZD11-KO bEnd.3 cells were generated using a GFP-containing CRISPR/Cas9 construct targeting exon 1 of the mouse PDZD11 gene (CRISPR sequence: GCCGGCCTATGAAAACCCTC) (3) (Fig. S1). GFP-positive single PDZD11-KO clones were sorted as described previously (3, 9) and screened by immunoblotting and IF microscopy. The genomic sequence of the PDZD11 locus was determined in distinct KO clones by PCR amplification and subcloning of a fragment surrounding the target before sequencing (3).

HeLa cells were grown in Dulbecco's modified Eagle's medium high glucose medium (Euroclone) supplemented with 10% fetal bovine serum (GIBCO), 100 U/ml penicillin (Euroclone), and 100 μg/ml streptomycin (Euroclone).

For transfection of mCCD cells, cells at 60% to 80% confluence were transfected 1 day after seeding, using jetOPTIMUS (Polyplus) following the manufacturer’s guidelines, and processed for IF 72 h later.

For transfection of HeLa cells, cells were seeded onto 13 mm diameter glass coverslips and transfected 12 h later (60–80% confluence) using the Ca^2+^ phosphate procedure (71). For each 13 mm coverslip, the transfection mix was prepared by adding 5 μl of 2.5 M CaCl_2_ (Sigma–Aldrich; catalog no.: # C-5080, stored at −20 °C until use) to 7.5 μg of total DNA (1.5 μg cytAEQ, 3 μg +3 μg combinations of control or experimental plasmids, see Figure legends) dissolved in Milli-Q H_2_O in a final volume of 50 μl. The solution was then mixed with 50 μl of Hepes-buffered solution 2X (280 mM NaCl, 50 mM Hepes, 1.5 mM Na_2_HPO_4_·(7H_2_O), pH 7.12, Sigma–Aldrich; catalog no.: # S9390, stored at −20 °C until use) and incubated 30 min at room temperature (RT). Just before transfection the growth medium of the cells was replaced with fresh medium, and the transfection mix was added to the cell monolayer, drop by drop. Eight hours later the medium was removed, and the cells were washed twice with Dulbecco’s PBS (D-PBS) (EuroClone; catalog no.: # ECB4004L) to remove excess of Ca^2+^ phosphate precipitates.

For cytosolic calcium measurements using the AEQ method, bEnd.3 and mCCD cells were plated into a 96-well plate (Corning; catalog no.: # 3610) at 50% to 60% of confluence and transduced for 48 h in their culture medium with adenovirus expressing cytosolic AEQ.

### Antibodies

The primary antibodies are listed in Table S1. They were used at the following dilutions for either immunoblotting or IF: rabbit anti-GFP (A-11122, Thermo Fisher Scientific, IB: 1/2000, IF: 1/200); mouse GFP (11814460001, Roche, IF: 1/100); mouse myc (9E10 (purified), in-house, IF: 1/100); mouse HA (32-6700, Thermo Fisher Scientific, IB: 1/1000); rabbit ß-catenin (C2206, Sigma, IB: 1/3500, IF: 1/500); goat VE-cadherin (sc-6458, Santa Cruz, IF: 1/1000); rat anti-ZO-1 (R40.76, a kind gift from Daniel Goodenough, Harvard Medical School, IF: 1:100); mouse anti-E-cadherin (610181, BD Biosciences, IF: 1/2500); mouse anti-β-tubulin (32-2600, Thermo Fisher Scientific, IB: 1/3500); mouse anti-PanPMCA (MA-3914 [clone 5F10], Thermo Fisher Scientific, IF: 1/1000, IB: 1/1000); rabbit anti-PLEKHA7 (Rb30388, in-house (2), IF: 1/1000); guinea pig anti-PLEKHA7 (GP2737, in-house (3), IF: 1/200); rabbit anti-PDZD11 (Rb29958 (3), IF: 1/50 to 1/100); and rat anti-Crb3a (1E6, a kind gift from André Le Bivic [IBDM, University of Marseille], IB: 1/1000).

Secondary antibodies (Table S1) for IF from Jackson ImmunoResearch were diluted at 1/300 and from Invitrogen at 1/100. Horseradish peroxidase–conjugated secondary antibodies used for IB (antimouse, antirat, and anti-rabbit were diluted 1/20000).

### Plasmids

Plasmids are described in Table S1.

### IF labeling

For IF labeling, mCCD cells were seeded either on 6.5 mm/0.4 μm pore polyester 24-well tissue culture inserts (Transwells, Corning Costar #3470) or on 12 mm glass coverslips in 24-well plates. bEnd.3 cells were plated on glass coverslips because they do not polarize. Cells (mCCD, bEnd.3) on coverslips were washed twice at RT with PBS before methanol (precooled at −80 °C) fixation during 8 min at −20 °C. Immunostaining was then performed as described previously (7), and mounting was done with Vectashield containing 4′,6-diamidino-2-phenylindole (VECTOR Laboratories). Cells (mCCD) grown on Transwell filters were fixed by 16 h incubation in methanol at −20 °C, followed by a 1 min treatment with acetone precooled at −20 °C, and then stained as described previously, using either two or three primary antibodies from different species and corresponding secondary antibodies in distinct fluorescence channels (7) (Table S1). Staining of nuclei with 4′,6-diamidino-2-phenylindole is colored in blue.

HeLa cells plated on 13 mm glass coverslips were fixed 48 h post-transfection for 20 min in a solution containing 3.7% (vol/vol) formaldehyde in D-PBS (formaldehyde stock solution 37% in H_2_O (Sigma–Aldrich; catalog: # F8775). Cells were then washed three times with D-PBS and permeabilized with 0.1% Triton X-100 BioChemica (PanReac AppliChem; catalog no.: # A1388) in PBS for 20 min, followed by washing twice for 15 min in 1% gelatine (type B from bovine skin) (Sigma–Aldrich; catalog no.: # G9382) in D-PBS at RT. The coverslips were then incubated for 90 min at 37  °C in a wet chamber with the specific primary antibodies diluted in D-PBS (anti-panPMCA and or anti-PDZD11). Staining was revealed by the incubation for 45 min at RT with the secondary fluorescent antibody AlexaFluor 488 and/or 594, respectively, diluted in D-PBS. After two washing steps of 15 min each, coverslips were mounted using Mowiol (Sigma–Aldrich; 81386-250G).

### IF microscopy

Slides were imaged on an upright microscope Leica 740 DM4B using 63X/NA 1.4 oil-immersion objective. Alternatively, confocal microscopy was done either with a Zeiss LSM800, or a Zeiss LSM700, or (for HeLa cells) a Leica SP5-TCS-II-RS inverted confocal microscopes using HCX Plan APO 63X/NA 1.4 oil-immersion objective and illumination with laser at the wavelength of 405, 488, 555, and 639 nm (Zeiss LSM) or 488 and 594 nm (Leica SP5-TCS-II-RS). For all images acquired with the Leica SP5-TCS-II-RS, scanning mode was XYZ, pinhole was set to 1 airy unit, format was 1024 × 1024, speed was 200 Hz, and pixel size was about 100 nm. To avoid crosstalk between fluorophores, sequential scans (scan between frames) were performed, and the beam splitter (dichroic) and the bandwidth were selected to make sure that the edges of the detection window are at least 10 nm apart from the excitation lines.

### Quantification of IF

Quantification of PanPMCA labeling at junctions and different domains of the plasma membrane of mCCD cells was carried out with FIJI software (fiji.sc) as described previously (3, 72), using ZO-1 as an internal reference for junctional labeling (not affected in PLEKHA7-KO and PDZD11-KO cells). For each replicate (*i.e.*, data point), five confocal images were analyzed. A cell–cell contact region was defined with the polyhedral tool of FIJI. Pixel intensity for PanPMCA channel and for ZO-1 (reference) channel was measured in the selected area, and the respective averaged background signal was subtracted for each channel. Relative signal was expressed as a ratio between PanPMCA and ZO-1, calculated over the five different images for each sample. Quantification of at least three biological repeats (corresponding to data points) was performed. To compare WT and PLEKHA7-KO cells, mixed cultures of WT and PLEKHA7-KO cells were used, distinguished by PLEKHA7 staining, to reduce variability. To compare WT and PDZD11-KO cells, separated cultures of WT and PDZD11-KO cells were used, keeping the acquisition parameters of the microscope strictly constant because immunostaining of endogenous PDZD11 is too heterogeneous to distinguish WT and KO cells with high confidence.

Quantification of PanPMCA distribution (zonular/lateral) at cell–cell contacts (XZ view) was done by calculating the zonular percentage of PanPMCA signal, which was obtained by dividing the integrated density of the signal in the zonular region, using ZO-1 to delimit the area (polygon selection tool of FIJI), by the integrated density of the signal at the entire cell–cell contact (PanPMCA staining along the overall cell–cell contact selected using the polygon selection tool of FIJI). About 1 to 5 cell–cell contacts from 3 to 11 different images from at least three distinct experiments were analyzed.

Quantification of PanPMCA labeling at plasma membrane of HeLa cells was carried out by FIJI software, by dividing the mean pixel fluorescence intensity within a defined region of interest tightly bordering the plasma membrane (selected using the freehand tool of FIJI) by the fluorescence intensity of an area of the same size either within the adjacent extracellular space or within the submembrane cytoplasm. Between 14 and 18 region of interest were measured for each experimental condition.

### Preparation of cell lysates and IB analysis

Cell lysates (500 μl per 10 cm dish) were obtained using radioimmunoprecipitation assay buffer (NaCl 150 mM, Tris-HCl 40 mM, pH 7.5, EDTA 2 mM, glycerol 10%, Triton X-100 1%, sodium deoxycholate 0.5%, SDS 0.2%) supplemented with protease inhibitor cocktail (Thermo Fisher Scientific; A32965). Lysates were sonicated (8 s at 66% amplitude with a Branson sonifier) and solubilized proteins were clarified by centrifugation (15 min at 4 °C, 13,000 rpm) (3, 7). Supernatants were mixed with SDS loading buffer and boiled 5 min at 95 °C before SDS-PAGE separation and Western blot done as described (7). For immunoblotting of PanPMCA, SDS loading buffer was supplemented with DTT and urea (final concentration of 100 mM and 2 M, respectively), and samples were warmed 10 min at 37 °C before loading onto gels. Enhanced chemiluminescence revelation was detected using Odyssey Imager (LI-COR). Numbers on the left of IBs (Figs 1*F*, 3*G*, 4*A*, *B*, *C*, *E*, *F*, and S2*B*) correspond to sizes in kilodalton. To compare PanPMCA expression between WT and KO cells (Fig. 1, *F* and *G*), quantification of densitometric signal was carried out in Image Studio Lite (LI-COR), using ß-tubulin signal for normalization, and calculated relative to the WT.

### Apical surface biotinylation

mCCD cells grown on 24 mm Transwells (Corning Costar #3450) were transfected using Lipofectamine 2000 (Thermo Fisher Scientific, 11668027) following the manufacturer’s instructions. Seventy-two hours after transfection, Pierce Cell Surface Protein Isolation Kit (Thermo Fisher Scientific; 89881) was used to biotinylate and isolate the apical surface proteins according to manufacturer’s instructions (see also (9)), using a lysis buffer composed of 150 mM NaCl, 20 mM Hepes (pH 7.4), 1 mM EDTA, and 1% Nonidet P-40 and complemented with protease inhibitor cocktail (Thermo Fisher Scientific; A32965). Elution from streptavidin beads was done with SDS loading buffer supplemented with 100 mM DTT and 2 M urea (incubation 30 min at RT, followed by 15 min at 37 °C). Samples were then analyzed by immunoblotting, along with inputs prepared in SDS buffer containing DTT and urea (15 min of incubation at 37 °C). Quantification of GFP(-PMCA4x/b) chemiluminescence signal was carried out in Image Studio Lite (LI-COR). For each genotype, densitometric signal of biotinylated GFP(-PMCA4x/b) was normalized to the input signal and calculated relative to the WT.

### RNA isolation and quantitative PCR

mRNA of confluent cells (mCCD and bEnd3) seeded in 35 mm dishes was extracted using NucleoSpin RNA Purification Kit from Macherey-Nagel (740955.50) and complementary DNA (cDNA) synthetized using iScript cDNA Synthesis Kit (Bio-Rad; 1708891), following the manufacturer’s instructions. Quantitative PCR analysis was performed on 20 ng of cDNA (each in triplicate) with SYBR Select Master Mix for CFX (Thermo Fisher Scientific; 4472942) for mouse PMCA1 (forward [Fw]: AGAAGTTCACCGTCATCAGG; reverse [Rv]: GTCACCGTACTTCACTTGGG), PMCA2 (Fw: TCCTCCTGGGACTCGAAGTT; Rv: AGTCGCTGTTGGTCATGTCA), PMCA3 (Fw: CTTTCCGTCCTTGGAGCTGAT; Rv: AGGCTAAGTGTGAACACCCC), a- and b-isoforms of PMCA4 (Fw: TACGGCACTTGGATGCTTGT; Rv: TAGTGAGTGCCCCCGATGTA), PMCA4a (Fw: CGGAAGCCCCCTTAAAGAGA; Rv: AGAGATGGAGGGGCAAGTTC), PMCA4b (Fw: ACTGAGGGAATGGACGAGAT; Rv: AGTTTGACGACTCTGATCTG), and GAPDH (internal standard (7), Fw: GTGCAGTGCCAGCCTCGTCC; Rv: CTCGGCCTTGACTGTGCCGT).

### Cytosolic calcium measurements using AEQ

For HeLa cells, 48 h after transfection cells were incubated (1 h, 37 °C) with the prosthetic group coelenterazine (5 μM, 1 h at 37 °C, Santa Cruz Biotechnology; catalog no.: sc-205904) in CaCl_2_-containing Krebs-Ringer buffer (KRB-CaCl_2_, 125 mM NaCl, 1 mM Na_3_PO_4_, 1 mM MgSO_4_, CaCl_2_ 1 mM, 5.5 mM glucose, 5 mM KCl, 20 mM HEPES, pH 7.4) and then transferred to the recording chamber, equipped with a perfusion system, located into the luminometer. For the measurements of [Ca^2+^]_cyt_, after a 20 s perfusion step with KRB-CaCl_2_, cells were stimulated with 100 μM histamine (Sigma, Cat# H7250) dissolved in KRB-CaCl_2_. The experiments were terminated by cell permeabilization with digitonin (100 μM, Sigma; catalog no.: D5628) in Ca^2+^-rich solution (10 mM CaCl_2_ in H_2_O). AEQ light emission was collected by means of an in-house built luminometer, equipped with a low-noise photomultiplier coupled by an A/D board to a computer-assisted acquisition system, with a 1 Hz sampling rate (71). This allowed the calibration of the recorded light signal to the total AEQ content. Conversion of light signal into Ca^2+^ concentration was performed as described in (30).

For bEnd.3 and mCCD cells, cells were incubated with coelenterazine as described previously, and cytosolic calcium measurements were carried out 48 h post-adenoviral transduction using a PerkinElmer EnVision plate reader equipped with two injector units. After AEQ reconstitution, cells were placed in 70 μl of KRB-CaCl_2_, and cytosolic Ca^2+^ transients were evoked by applying 100 μM ATP (Amersham Biosciences; catalog no.: 27-1006-03) in the presence of 20 μM Tert-butylhydroquinone (Sigma; catalog no.: # 112976), to inhibit Ca^2+^ reuptake by the SERCA. The experiment was concluded by the injection of a hypotonic, Ca^2+^-rich, digitonin containing solution to discharge the reconstituted AEQ and permit the calibration protocol (30). Output data were analyzed and calibrated with a custom-made macroenabled Excel workbook.

### Single-cell Fura-2 measurement of endogenous PMCA-mediated calcium extrusion

bEnd.3 cells were plated at a density of 100,000 cells/cm^2^ in 35 mm FluoroDishes (Optical quality glass bottom, WPI, FD35-100) 48 h before analysis. Cells were loaded with Fura-2 (Thermo Fisher Scientific; F1225) by removing the medium, washing twice with calcium medium for imaging (CMI) (N-methyl-D-glucamine, Thermo Fisher Scientific; 126841000, 140 mM KCl, 5 mM MgCl_2_, 1 mM, Hepes 20 mM, pH 7.4, glucose 10 mM, CaCl_2_ 2 mM), and incubating in CMI containing Pluronic F-127 (Thermo Fisher Scientific; P3000MP) 0.02% and Fura-2 AM 2 μM for 30 min at RT in the dark. Cells were then washed with CMI and incubated in CMI for 10 min at RT in the dark. The analysis was carried out at RT, within 60 min following the loading. Single-cell imaging of 10 to 20 cells was carried out with a Nikon Eclipse Calcium BioFlux microscope using MetaFluor imaging software (Molecular Devices). Time-lapse was performed every 2 s, switching between 340 and 380 nm as excitation wavelengths, the emission being measured at 510 nm. After measuring cytosolic calcium concentration at steady-state during 2 minutes, thapsigargin (Thermo Fisher Scientific; T7458) (final concentration 1 μM) was added to irreversibly block SERCA activity. When cytosolic calcium concentration reached a plateau, the CMI was removed and replaced with CMI containing 0 mM CaCl_2_ and 1 mM EGTA, resulting in calcium extrusion through the plasma membrane.

### Analysis of calcium measurement data

GraphPad Prism 8 (GraphPad Software) was used to fit a one-phase exponential decay to the decrease of calcium transients for the normalized peaks. Time = 0 s corresponds to the calcium switch instant. Range of fitting was 0 to 200 s for Fura-2, 0 to 30 s for AEQ for bEnd.3 and mCCD cells, and 0 to 70 s for AEQ for HeLa cells. Extrusion rate was estimated by the time constant τ of the exponential decay (32). For Ca^2+^ measurement with AEQ probe, each independent transfection or transduction was considered as a ‘biological replicate’ and each coverslip or each well is considered as a technical replicate within the same transfection or transduction. The respective numbers for biological replicate (independent transfection or transduction) or technical replicate (n) are indicated in the Figure legends and/or in the text.

### Recombinant protein expression and GST pull downs

Production and normalization of GST-tagged fusion proteins (baits) was performed as described previously (7). Preys and additional proteins were expressed in HEK293T cells (2.3 × 10^6^ cells per 10 cm dish) transfected with 10 μg of DNA using PEI (Polysciences; # 23966-2). About 48 to 72 h later, after washing with PBS, cells were lysed in 500 μl coimmunoprecipitation buffer (NaCl 150 mM, Tris–HCl 20 mM, pH 7.5, Nonidet P-40 1%, EDTA 1 mM) supplemented with protease inhibitor cocktail (Thermo Fisher Scientific; A32965). Lysates were sonicated (8 s at 66%, Branson sonifier) and centrifuged (15 min at 13,000 rpm, at 4 °C). The supernatant was kept and the pellet resuspended in 50 μl of SDS buffer (SDS 1%, Tris–HCl 10 mM, pH 7.5, EDTA 2 mM, DTT 0.5 mM, PMSF 0.5 mM), sonicated 3 s at 15%, incubated at 100 °C for 5 min, clarified by centrifugation, brought to a volume of 0.5 ml with coimmunoprecipitation buffer, and mixed with the first supernatant collected (3, 7). Prey and additional protein loadings were normalized by immunoblotting.

For GST pull downs, 5 μg of GST-fusion protein bait were coupled to Pierce Glutathione Magnetic Agarose Beads (Thermo Fisher Scientific; #78602) before overnight incubation at 4 °C with normalized HEK293T lysates of preys and additional (third) proteins (7). Proteins bound to the beads were eluted with SDS loading buffer supplemented with DTT and urea (final concentration of 100 mM and 2 M, respectively) warmed 15 min at 37 °C, before analysis by immunoblotting.

Quantification of GFP-PMCA4x/b chemiluminescence signal intensity in GST-PLEKHA7_ww-PH_ pull downs in the presence of PDZD11-HA or CFP-HA was carried out in Image Studio Lite (LI-COR), normalized to bait signal (Ponceau S straining), and calculated relative to the value in the absence of additional protein.

### Statistical analysis

Statistical significance was determined using GraphPad Prism 8 software; sample size (n), *p*-values (p), and statistical tests performed are specified in Figure legends. Values are shown as mean ± SD. Significance corresponds to ∗*p <* 0.05, ∗∗ or °°*p* < 0.01, ∗∗∗*p <* 0.001, ∗∗∗∗*p* < 0.0001. Likely outliers were removed by ROUT method, and either mixed-effects analysis or repeated measures one-way ANOVA were used for multiple comparisons, before Dunnett’s test to compare every mean to control mean or Sidak’s test for multiple pairwise comparisons. Student’s *t* test was used to compare WT results to those of either PLEKHA7-KO or PDZD11-KO cells. For Fura-2-based Ca^2+^ measurement experiments statistical significance was determined by one-way ANOVA before Dunnett’s test. For AEQ-based Ca^2+^ measurement experiments, likely outliers were removed by ROUT method and statistical significance was determined by Student’s *t* test to compare WT results to P11-KO mCCD, one-way ANOVA before Dunnett’s test for bEnd.3 cells to compare every mean to control mean or one-way ANOVA before Tukey’s multiple comparison test for HeLa cells. Additional statistical data are provided in Table S2 (Table S2).

## Data availability

Data and materials are available from the corresponding authors (S. C. and M. B.), upon request.

## Supporting information

This article contains supporting information (2, 3, 7, 9, 26, 30, 31, 58, 73, 74, 75, 76).

## Conflict of interest

The authors declare that they have no conflicts of interest with the contents of this article.
