# Supplementary material for: The PLEKHA7–PDZD11 complex regulates the localization of the calcium pump PMCA and calcium handling in cultured cells
Source: J Biol Chem. 2022 Jun 15;298(8):102138. doi: 10.1016/j.jbc.2022.102138 (PMC9307954; doi:10.1016/j.jbc.2022.102138)
Supplement: Table S1 [file mmc1.docx]

**Supporting Information Table 1**. Key Resources.

| **REAGENT or RESOURCE** | **SOURCE** | **IDENTIFIER** |
| --- | --- | --- |
| **Antibodies** | | |
| Rabbit polyclonal anti-PLEKHA7 | Citi Laboratory (1) | Rb30388 |
| Guinea pig polyclonal anti-PLEKHA7 | Citi Laboratory (2) | GP2737 |
| Rabbit polyclonal anti-PDZD11 | Citi Laboratory (2) | Rb29958 |
| Mouse monoclonal anti-β-tubulin | Thermo Fisher Scientific | Cat# 32-2600,  RRID: AB_2533072 |
| Rabbit polyclonal anti-GFP | Thermo Fisher Scientific | Cat# A-11122,  RRID: AB_221569 |
| Mouse monoclonal anti-GFP | Roche | Cat# 11814460001, RRID: AB_390913 |
| Mouse monoclonal anti-HA | Thermo Fisher Scientific | Cat# 32-6700,  RRID: AB_2533092 |
| Mouse monoclonal anti-myc | Citi Laboratory | 9E10 |
| Mouse monoclonal anti-E-cadherin | BD Biosciences | Cat# 610181,  RRID: AB_397580 |
| Goat polyclonal anti-VE-cadherin | Santa Cruz Biotechnology | Cat# sc-6458,  RRID: AB_2077955 |
| Rabbit polyclonal anti-β-catenin | Sigma-Aldrich | Cat# C2206,  RRID: AB_476831 |
| Rat monoclonal anti-ZO-1 | Goodenough Laboratory (Harvard Medical School) | R40.76,  RRID: AB_2205518 |
| Mouse monoclonal anti-PanPMCA (5F10) | Thermo Fisher Scientific | Cat# MA-3914,  RRID: AB_2061566 |
| Rat monoclonal anti-Crb3a | Gift from A. Le Bivic (University of Marseille, France) | 1E6 |
| Alexa Fluor 488-AffiniPure Donkey anti-Rabbit IgG | Jackson ImmunoResearch | Cat# 711-545-152, RRID: AB_2313584 |
| Alexa Fluor 488-AffiniPure Donkey anti-Mouse IgG | Jackson ImmunoResearch | Cat# 715-546-151, RRID: AB_2340850 |
| Alexa Fluor 488-AffiniPure Donkey anti-Guinea Pig IgG | Jackson ImmunoResearch | Cat# 706-546-148, RRID: AB_2340473 |
| Alexa Fluor 594-AffiniPure Goat anti-Rabbit IgG | Invitrogen | Cat# A11012,  RRID: AB_2534079 |
| Alexa Fluor 488-AffiniPure Goat anti-Mouse | Invitrogen | Cat# A11001,  RRID: AB_2534069 |
| Cy3-AffiniPure Donkey anti-Mouse IgG | Jackson ImmunoResearch | Cat# 715-165-151, RRID: AB_2315777 |
| Cy3-AffiniPure Donkey anti-Rabbit IgG | Jackson ImmunoResearch | Cat# 711-165-152, RRID: AB_2307443 |
| Cy5-AffiniPure Donkey anti-Rat IgG | Jackson ImmunoResearch | Cat# 712-175-153, RRID: AB_2340672 |
| Alexa Fluor 647-AffiniPure Donkey anti-Goat | Jackson ImmunoResearch | Cat# 705-606-147, RRID: AB_2340438 |
| Anti-Rat IgG (H+L), HRP conjugate | Thermo Fisher Scientific | Cat# 62–9520,  RRID: AB_2533965 |
| Anti-Rabbit IgG (H+L), HRP conjugate | Promega | Cat# W4011,  RRID: AB_430833 |
| Anti-Mouse IgG (H+L), HRP conjugate | Promega | Cat# W4021,  RRID: AB_430834 |
| **Bacterial Strains** | | |
| DH5-α Competent cells | Thermo Fisher Scientific | Cat# 18265017 |
| BL21-DE3 Competent cells | NEB | Cat# C2530H |
| **Chemicals and Recombinant Proteins** | | |
| Adenosine-5’-triphosphate (ATP) | Amersham Biosciences | Cat# 27-1006-03 |
| Coelenterazine | Santa Cruz Biotechnology | Cat# sc-205904 |
| Digitonin | Sigma | Cat# D5628 |
| Fura-2 AM | Thermo Fisher Scientific | Cat# F1225 |
| Histamine | Sigma | Cat# H7250 |
| N-Methyl-D-glucamine (NMDG) | Thermo Fisher Scientific | Cat# 126841000 |
| Pluronic F-127 | Thermo Fisher Scientific | Cat# P3000MP |
| Tert-butylhydroquinone (TBHQ) | Sigma | Cat# 112976 |
| Thapsigargin | Thermo Fisher Scientific | Cat# T7458 |
| GST- human PLEKHA7-WW-PH (1-284) | Citi Laboratory (2) | S1793 |
| **Critical Commercial Assays** | | |
| Pierce Cell Surface Protein Isolation Kit | Thermo Fisher Scientific | Cat# 89881 |
| Lipofectamine 2000 | Invitrogen | Cat# 11668027 |
| jetOPTIMUS DNA Transfection Reagent | Polyplus | Cat# 117-15 |
| Polyethylenimine, Linear, MW 25000 | Polysciences | Cat# 23966-2 |
| Pierce Glutathione Magnetic Agarose Beads | Thermo Fisher Scientific | Cat# 78602 |
| NucleoSpin RNA Purification Kit | Macherey-Nagel | Cat# 740955.50 |
| iScript cDNA Synthesis Kit | Bio-Rad | Cat# 1708891 |
| SYBR Select Master Mix for CFX | Thermo Fisher Scientific (Life Technologies) | Cat# 4472942 |
| DNeasy Blood and Tissue kit | QIAGEN | Cat# 69504 |
| Transwells (6.5 mm inserts, 0.4 µm pore size, polyester membrane) | Corning Costar | Cat# 3470 |
| Transwells (24 mm inserts, 0.4 µm pore size, polyester membrane) | Corning Costar | Cat# 3450 |
| 35-mm FluoroDishes (Optical quality glass bottom) | WPI | Cat# FD35-100 |
| Protease inhibitor cocktail | Thermo Fisher Scientific | Cat# A32965 |
| **Experimental Models: Cell Lines** | | |
| Mouse cortical collecting duct cell line, mCCD WT N64-Tet-ON | Feraille Laboratory (UniGe) | N/A |
| Mouse cortical collecting duct cell line, mCCD PLEKHA7-KO N64-Tet-ON | Citi Laboratory (2,3) | N/A |
| Mouse cortical collecting duct cell line, mCCD PDZD11-KO N64-Tet-ON | Citi Laboratory (2) | N/A |
| Mouse brain microvascular endothelial (endothelioma) cell line, bEnd.3 WT | Imhof Laboratory, Unige | N/A |
| Mouse brain microvascular endothelial (endothelioma) cell line, bEnd.3 PDZD11-KO | Citi Laboratory (This paper) | N/A |
| **Plasmids** | | |
| EGFP-hPMCA4x/b in pEGFP-C2 | Strehler Laboratory (Mayo Clinic) (4) | S2140 |
| GFP-hPMCA4x/b ∆PDZ-binding (1-1199) in pcDNA3.1(-) | Citi Laboratory (This paper, based on (5)) | S2401 |
| GFP-myc in pcDNA3.1(-) | Citi Laboratory (6) | S1166 |
| pcDNA3.1(+) (control) | Brini Laboratory | N/A |
| hPLEKHA7-myc-His in pCDNA3.1 | Citi Laboratory  (7) | S1233 |
| hPDZD11-HA in pcDNA3.1(zeo+) | Citi Laboratory (2) | S1766 |
| CFP-HA in pcDNA3.1(zeo+) | Citi Laboratory (2) | S1150 |
| cytAEQ in VR1012 | Brini Laboratory  (8) | NA |
| PMCA4x/b in pSG5 | (9,10) | N/A |
| PMCA4x/a in pcDNA3.0 | (9,10) | N/A |
| mouse PDZD11 exon 1 CRISPR target (sequence: GCCGGCCTATGAAAACCCTC) in pSpCas9(BB)-2A-GFP (px458) | Citi Laboratory (2) | S1768 |
| GFP-myc in pTRE2hyg | Citi Laboratory (7) | S1210 |
| **Oligonucleotides** | | |
| Genotyping primers: mouse PDZD11  Fw: tgaGCGGCCGCGGCCCTGAGTTAAGTGGTCG  Rv: tgaGGATCCGGGCAGAAACTGGGTCAACT | Citi Laboratory (2) | N/A |
| qPCR mouse PMCA1  Fw: AGAAGTTCACCGTCATCAGG  Rv: GTCACCGTACTTCACTTGGG | Citi Laboratory (This paper) | N/A |
| qPCR mouse PMCA2  Fw: TCCTCCTGGGACTCGAAGTT  Rv: AGTCGCTGTTGGTCATGTCA | Citi Laboratory (This paper) | N/A |
| qPCR mouse PMCA3  Fw: CTTTCCGTCCTTGGAGCTGAT  Rv: AGGCTAAGTGTGAACACCCC | Citi Laboratory (This paper) | N/A |
| qPCR mouse PanPMCA4x  Fw: TACGGCACTTGGATGCTTGT  Rv: TAGTGAGTGCCCCCGATGTA | Citi Laboratory (This paper) | N/A |
| qPCR mouse PMCA4x/a  Fw: CGGAAGCCCCCTTAAAGAGA  Rv: AGAGATGGAGGGGCAAGTTC | Citi Laboratory (This paper) | N/A |
| qPCR mouse PMCA4x/b  Fw: ACTGAGGGAATGGACGAGAT  Rv: AGTTTGACGACTCTGATCTG | Citi Laboratory (This paper) | N/A |
| qPCR mouse GAPDH  Fw: GTGCAGTGCCAGCCTCGTCC  Rv: CTCGGCCTTGACTGTGCCGT | Citi Laboratory (11) | N/A |
| **Softwares and Algorithms** | | |
| FIJI | (12) | imagej.net/Fiji |
| Adobe Photoshop | N/A | adobe.com |
| Adobe Illustrator | N/A | adobe.com |
| Image Studio Lite | LI-COR | www.licor.com/bio/image-studio-lite/ |
| SnapGene | N/A | snapgene.com/ |
| GraphPad Prism 8 | N/A | graphpad.com/scientific-software/prism/ |

REFERENCES

1. Pulimeno, P., Bauer, C., Stutz, J., and Citi, S. (2010) PLEKHA7 Is an Adherens Junction Protein with a Tissue Distribution and Subcellular Localization Distinct from ZO-1 and E-Cadherin. *PLoS One* **5**, 10.1371/journal.pone.0012207

2. Guerrera, D., Shah, J., Vasileva, E., Sluysmans, S., Mean, I., Jond, L., Poser, I., Mann, M., Hyman, A. A., and Citi, S. (2016) PLEKHA7 Recruits PDZD11 to Adherens Junctions to Stabilize Nectins. *J Biol Chem* **291**, 11016-11029

3. Shah, J., Rouaud, F., Guerrera, D., Vasileva, E., Popov, L. M., Kelley, W. L., Rubinstein, E., Carette, J. E., Amieva, M. R., and Citi, S. (2018) A Dock-and-Lock Mechanism Clusters ADAM10 at Cell-Cell Junctions to Promote alpha-Toxin Cytotoxicity. *Cell Rep* **25**, 2132-2147 e2137

4. Chicka, M. C., and Strehler, E. E. (2003) Alternative splicing of the first intracellular loop of plasma membrane Ca2+-ATPase isoform 2 alters its membrane targeting. *J Biol Chem* **278**, 18464-18470

5. Goellner, G. M., DeMarco, S. J., and Strehler, E. E. (2003) Characterization of PISP, a novel single-PDZ protein that binds to all plasma membrane Ca2+-ATPase b-splice variants. *Ann N Y Acad Sci* **986**, 461-471

6. Paschoud, S., Yu, D., Pulimeno, P., Jond, L., Turner, J. R., and Citi, S. (2011) Cingulin and paracingulin show similar dynamic behaviour, but are recruited independently to junctions. *Molecular membrane biology* **28**, 123-135

7. Paschoud, S., Jond, L., Guerrera, D., and Citi, S. (2014) PLEKHA7 modulates epithelial tight junction barrier function. *Tissue barriers* **2**, e28755

8. Brini, M., Marsault, R., Bastianutto, C., Alvarez, J., Pozzan, T., and Rizzuto, R. (1995) Transfected aequorin in the measurement of cytosolic Ca2+ concentration ([Ca2+]c). A critical evaluation. *J Biol Chem* **270**, 9896-9903

9. Brini, M., Coletto, L., Pierobon, N., Kraev, N., Guerini, D., and Carafoli, E. (2003) A comparative functional analysis of plasma membrane Ca2+ pump isoforms in intact cells. *J Biol Chem* **278**, 24500-24508

10. Preiano, B. S., Guerini, D., and Carafoli, E. (1996) Expression and functional characterization of isoforms 4 of the plasma membrane calcium pump. *Biochemistry* **35**, 7946-7953

11. Sluysmans, S., Mean, I., Xiao, T., Boukhatemi, A., Ferreira, F., Jond, L., Mutero, A., Chang, C. J., and Citi, S. (2021) PLEKHA5, PLEKHA6 and PLEKHA7 bind to PDZD11 to target the Menkes ATPase ATP7A to the cell periphery and regulate copper homeostasis. *Mol Biol Cell* **32**, <https://doi.org/10.1091/mbc.E1021-1007-0355>

12. Schindelin, J., Arganda-Carreras, I., Frise, E., Kaynig, V., Longair, M., Pietzsch, T., Preibisch, S., Rueden, C., Saalfeld, S., Schmid, B., Tinevez, J. Y., White, D. J., Hartenstein, V., Eliceiri, K., Tomancak, P., and Cardona, A. (2012) Fiji: an open-source platform for biological-image analysis. *Nature methods* **9**, 676-682
