# Supplementary material for: The PLEKHA7–PDZD11 complex regulates the localization of the calcium pump PMCA and calcium handling in cultured cells
Source: J Biol Chem. 2022 Jun 15;298(8):102138. doi: 10.1016/j.jbc.2022.102138 (PMC9307954; doi:10.1016/j.jbc.2022.102138)
Supplement: Table S2 [file mmc2.docx]

**Supporting Information Table 2.** Tukey’s post-hoc test for full data for Figure 7D and 7E.

| **Tukey's multiple comparisons test, figure 7D** |  | **Adjusted**  **P Value** |
| --- | --- | --- |
| CTRL vs. P11 | ** | 0.0093 |
| CTRL vs. P7 | ns | >0.9999 |
| CTRL vs. PMCA4x/b | **** | <0.0001 |
| CTRL vs. PMCA4x/b + P11 | ns | 0.7750 |
| CTRL vs. PMCA4x/a | **** | <0.0001 |
| CTRL vs. PMCA4x/a + P11 | **** | <0.0001 |
| CTRL vs. PMCA4x/b + P7 | **** | <0.0001 |
| P11 vs. P7 | * | 0.0158 |
| P11 vs. PMCA4x/b | **** | <0.0001 |
| P11 vs. PMCA4x/b + P11 | *** | 0.0005 |
| P11 vs. PMCA4x/a | **** | <0.0001 |
| P11 vs. PMCA4x/a + P11 | **** | <0.0001 |
| P11 vs. PMCA4x/b + P7 | **** | <0.0001 |
| P7 vs. PMCA4x/b | **** | <0.0001 |
| P7 vs. PMCA4x/b + P11 | ns | 0.9592 |
| P7 vs. PMCA4x/a | **** | <0.0001 |
| P7 vs. PMCA4x/a + P11 | **** | <0.0001 |
| P7 vs. PMCA4x/b + P7 | **** | <0.0001 |
| PMCA4x/b vs. PMCA4x/b + P11 | ** | 0.0010 |
| PMCA4x/b vs. PMCA4x/a | **** | <0.0001 |
| PMCA4x/b vs. PMCA4x/a + P11 | **** | <0.0001 |
| PMCA4x/b vs. PMCA4x/b + P7 | ns | 0.9780 |
| PMCA4x/b + P11 vs. PMCA4x/a | **** | <0.0001 |
| PMCA4x/b + P11 vs. PMCA4x/a + P11 | **** | <0.0001 |
| PMCA4x/b + P11 vs. PMCA4x/b + P7 | *** | 0.0006 |
| PMCA4x/a vs. PMCA4x/a + P11 | ns | 0.9691 |
| PMCA4x/a vs. PMCA4x/b + P7 | **** | <0.0001 |
| PMCA4x/a + P11 vs. PMCA4x/b + P7 | *** | 0.0002 |

| **Tukey's multiple comparisons test, figure 7E** |  | **Adjusted**  **P Value** |
| --- | --- | --- |
| CTRL vs. P11 | ns | 0.6050 |
| CTRL vs. P7 | **** | <0.0001 |
| CTRL vs. PMCA4x/b | ns | 0.9086 |
| CTRL vs. PMCA4x/b + P11 | ns | 0.1413 |
| CTRL vs. PMCA4x/a | ** | 0.0039 |
| CTRL vs. PMCA4x/a + P11 | **** | <0.0001 |
| CTRL vs. PMCA4x/b + P7 | ns | 0.9997 |
| P11 vs. P7 | ns | 0.1032 |
| P11 vs. PMCA4x/b | ns | 0.1138 |
| P11 vs. PMCA4x/b + P11 | ** | 0.0054 |
| P11 vs. PMCA4x/a | *** | 0.0001 |
| P11 vs. PMCA4x/a + P11 | **** | <0.0001 |
| P11 vs. PMCA4x/b + P7 | ns | 0.9593 |
| P7 vs. PMCA4x/b | **** | <0.0001 |
| P7 vs. PMCA4x/b + P11 | **** | <0.0001 |
| P7 vs. PMCA4x/a | **** | <0.0001 |
| P7 vs. PMCA4x/a + P11 | **** | <0.0001 |
| P7 vs. PMCA4x/b + P7 | ** | 0.0035 |
| PMCA4x/b vs. PMCA4x/b + P11 | ns | 0.7743 |
| PMCA4x/b vs. PMCA4x/a | ns | 0.0578 |
| PMCA4x/b vs. PMCA4x/a + P11 | **** | <0.0001 |
| PMCA4x/b vs. PMCA4x/b + P7 | ns | 0.8377 |
| PMCA4x/b + P11 vs. PMCA4x/a | ns | 0.6910 |
| PMCA4x/b + P11 vs. PMCA4x/a + P11 | * | 0.0332 |
| PMCA4x/b + P11 vs. PMCA4x/b + P7 | ns | 0.1697 |
| PMCA4x/a vs. PMCA4x/a + P11 | ns | 0.9667 |
| PMCA4x/a vs. PMCA4x/b + P7 | ** | 0.0061 |
| PMCA4x/a + P11 vs. PMCA4x/b + P7 | **** | <0.0001 |
