# Supplementary material for: The PLEKHA7–PDZD11 complex regulates the localization of the calcium pump PMCA and calcium handling in cultured cells
Source: J Biol Chem. 2022 Jun 15;298(8):102138. doi: 10.1016/j.jbc.2022.102138 (PMC9307954; doi:10.1016/j.jbc.2022.102138)
Supplement: Table S3 [file mmc3.docx]

**Table S3. Two-way ANOVA on peaks: CTRL/P11/P7 vs CTRL/PMCA4x/b**

| Two-way ANOVA | Ordinary |  |  |  |  |
| --- | --- | --- | --- | --- | --- |
| Alpha | 0.05 |  |  |  |  |
|  |  |  |  |  |  |
| Source of Variation | P value | P value summary |  |  |  |
| Interaction | 0.7806 | ns |  |  |  |
| CTRL/P11/P7 | <0.0001 | **** |  |  |  |
| CTRL/PMCA4x/b | <0.0001 | **** |  |  |  |
|  |  |  |  |  |  |
| ANOVA table | SS (Type III) | DF | MS | F (DFn, DFd) | P value |
| Interaction | 0.03104 | 2 | 0.01552 | F (2, 142) = 0.2481 | P=0.7806 |
| CTRL/P11/P7 | 2.219 | 2 | 1.110 | F (2, 142) = 17.74 | P<0.0001 |
| CTRL/PMCA4x/b | 4.963 | 1 | 4.963 | F (1, 142) = 79.33 | P<0.0001 |
| Residual | 8.884 | 142 | 0.06256 |  |  |

| Number of families | 1 |  |
| --- | --- | --- |
| Number of comparisons per family | 15 |  |
| Alpha | 0.05 |  |
|  |  |  |
| Sidak's multiple comparisons test | Summary | Adjusted P Value |
|  |  |  |
| P11:PMCA4x/b vs. P11:CTRL | *** | 0.0006 |
| P11:PMCA4x/b vs. CTRL:PMCA4x/b | ** | 0.0013 |
| P11:PMCA4x/b vs. CTRL:CTRL | ns | 0.8940 |
| P11:PMCA4x/b vs. P7:PMCA4x/b | *** | 0.0008 |
| P11:PMCA4x/b vs. P7:CTRL | ns | 0.9954 |
| P11:CTRL vs. CTRL:PMCA4x/b | **** | <0.0001 |
| P11:CTRL vs. CTRL:CTRL | * | 0.0108 |
| P11:CTRL vs. P7:PMCA4x/b | **** | <0.0001 |
| P11:CTRL vs. P7:CTRL | * | 0.0180 |
| CTRL:PMCA4x/b vs. CTRL:CTRL | **** | <0.0001 |
| CTRL:PMCA4x/b vs. P7:PMCA4x/b | ns | 0.9986 |
| CTRL:PMCA4x/b vs. P7:CTRL | **** | <0.0001 |
| CTRL:CTRL vs. P7:PMCA4x/b | **** | <0.0001 |
| CTRL:CTRL vs. P7:CTRL | ns | >0.9999 |
| P7:PMCA4x/b vs. P7:CTRL | **** | <0.0001 |

**Two-way ANOVA on tau: CTRL/P11/P7 vs CTRL/PMCA4x/b**

| Two-way ANOVA | Ordinary |  |  |  |  |
| --- | --- | --- | --- | --- | --- |
| Alpha | 0.05 |  |  |  |  |
|  |  |  |  |  |  |
| Source of Variation | P value | P value summary |  |  |  |
| Interaction | 0.0174 | * |  |  |  |
| CTRL/P11/P7 | <0.0001 | **** |  |  |  |
| CTRL/PMCA4x/b | <0.0001 | **** |  |  |  |
|  |  |  |  |  |  |
| ANOVA table | SS (Type III) | DF | MS | F (DFn, DFd) | P value |
| Interaction | 19.45 | 2 | 9.723 | F (2, 139) = 4.169 | P=0.0174 |
| CTRL/P11/P7 | 50.70 | 2 | 25.35 | F (2, 139) = 10.87 | P<0.0001 |
| CTRL/PMCA4x/b | 63.71 | 1 | 63.71 | F (1, 139) = 27.32 | P<0.0001 |
| Residual | 324.2 | 139 | 2.332 |  |  |

| Number of families | 1 |  |
| --- | --- | --- |
| Number of comparisons per family | 15 |  |
| Alpha | 0.05 |  |
|  |  |  |
| Sidak's multiple comparisons test | Summary | Adjusted P Value |
|  |  |  |
| P11:PMCA4x/b vs. P11:CTRL | ** | 0.0059 |
| P11:PMCA4x/b vs. CTRL:PMCA4x/b | ns | 0.8891 |
| P11:PMCA4x/b vs. CTRL:CTRL | ns | 0.1588 |
| P11:PMCA4x/b vs. P7:PMCA4x/b | ns | 0.1928 |
| P11:PMCA4x/b vs. P7:CTRL | **** | <0.0001 |
| P11:CTRL vs. CTRL:PMCA4x/b | ns | 0.1264 |
| P11:CTRL vs. CTRL:CTRL | ns | 0.7216 |
| P11:CTRL vs. P7:PMCA4x/b | ns | 0.9952 |
| P11:CTRL vs. P7:CTRL | ns | 0.1141 |
| CTRL:PMCA4x/b vs. CTRL:CTRL | ns | 0.9778 |
| CTRL:PMCA4x/b vs. P7:PMCA4x/b | ns | 0.9374 |
| CTRL:PMCA4x/b vs. P7:CTRL | **** | <0.0001 |
| CTRL:CTRL vs. P7:PMCA4x/b | ns | >0.9999 |
| CTRL:CTRL vs. P7:CTRL | **** | <0.0001 |
| P7:PMCA4x/b vs. P7:CTRL | ** | 0.0039 |
