# Supplementary material for: The PLEKHA7–PDZD11 complex regulates the localization of the calcium pump PMCA and calcium handling in cultured cells
Source: J Biol Chem. 2022 Jun 15;298(8):102138. doi: 10.1016/j.jbc.2022.102138 (PMC9307954; doi:10.1016/j.jbc.2022.102138)
Supplement: Figure S1 [file mmc4.pdf]

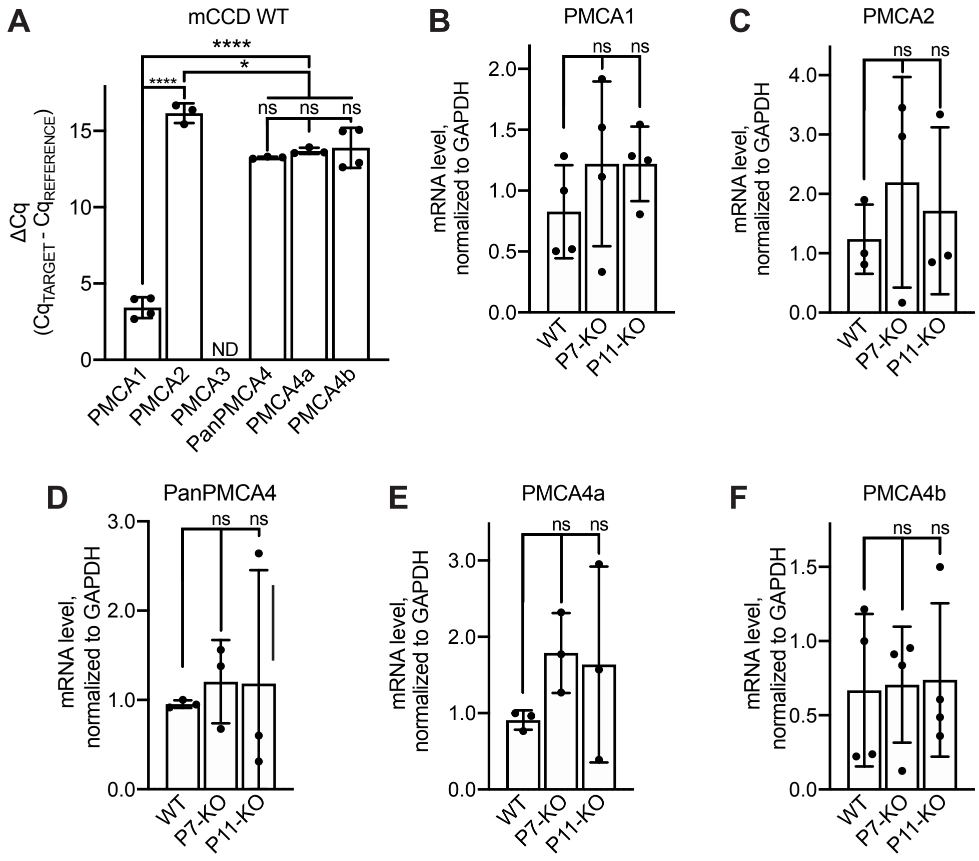

Figure S1 (Related to Figures 1, 2, 3, 7). mRNA levels of PMCA isoforms in mCCD cells.

(A) mRNA expression profiles of PMCA isoforms in mCCD WT as determined by qRT-PCR. Quantification cycle (Cq) values of PMCA isoforms are normalized to GAPDH (ΔCq). The closer the ΔCq is to zero, the more the isoform is expressed, showing the following expression order: PMCA1 > PMCA4 > PMCA2 (PMCA3 is not detected (ND)). Dots show replicates (n=3-4), and bars represent mean and SD. One-way ANOVA with post hoc Sidak's multiple comparisons test (*$p<0.05$, ****$p<0.0001$, ns: not significant).

(B-F) Quantification by qRT-PCR of the mRNA levels of PMCA1 (B), PMCA2 (C), PanPMCA4 (all isoforms) (D), PMCA4a (E) and PMCA4b (F) in WT, PLEKHA7-KO (P7-KO) and PDZD11-KO (P11-KO) mCCD cells (using GAPDH as internal standard), relative to WT cells. Dots show replicates (n=3-4), and bars represent mean and SD. One-way ANOVA with post hoc Dunnett's test (ns: not significant).
