# Supplementary material for: The PLEKHA7–PDZD11 complex regulates the localization of the calcium pump PMCA and calcium handling in cultured cells
Source: J Biol Chem. 2022 Jun 15;298(8):102138. doi: 10.1016/j.jbc.2022.102138 (PMC9307954; doi:10.1016/j.jbc.2022.102138)
Supplement: Figure S2 [file mmc5.pdf]

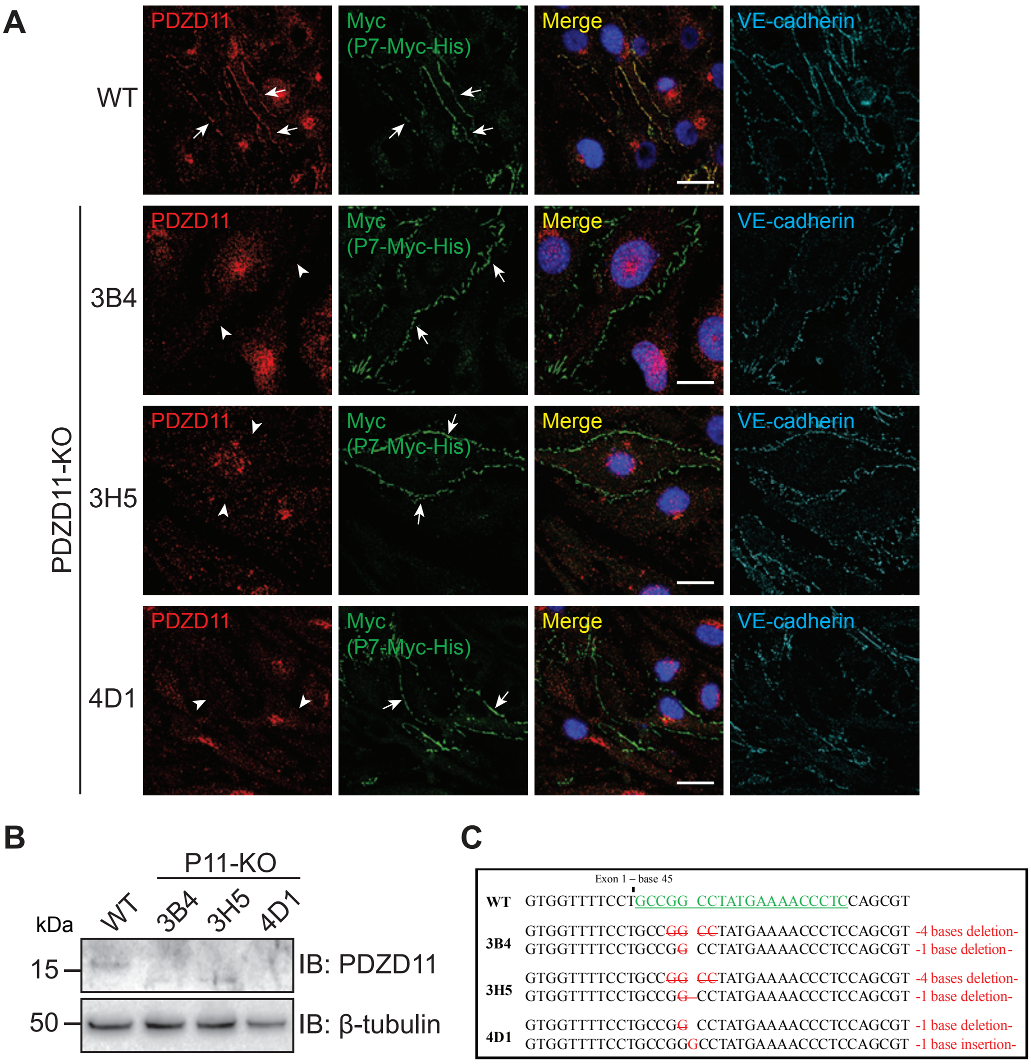

Figure S2 (related to Figure 7). Generation of PDZD11-KO bEnd.3 cell clones.
(A-B) Validation of CRISPR/Cas9-mediated deletion of PDZD11 (P11) in bEnd.3 by IF microscopy (A) and IB (B) analysis. Exogenous myc-tagged PLEKHA7 was transfected into the cells to increase the junctional localization of PDZD11 for detection by IF microscopy (Guerrera et al, JBC 2016), since endogenous PDZD11 labeling is weak or undetectable. VE-cadherin was used as a junctional marker for internal reference in IF analysis. Arrows indicate labeling, arrowheads indicate undetectable labeling. Bars= 20 μm. (C) Genomic sequencing of the PDZD11-KO bEnd.3 clones. CRISPR target is depicted in green in the WT sequence, with its position in the exon, and respective indels in the alleles of the KO clones obtained are indicated in red.
