# Supplementary material for: The PLEKHA7–PDZD11 complex regulates the localization of the calcium pump PMCA and calcium handling in cultured cells
Source: J Biol Chem. 2022 Jun 15;298(8):102138. doi: 10.1016/j.jbc.2022.102138 (PMC9307954; doi:10.1016/j.jbc.2022.102138)
Supplement: Figure S3 [file mmc6.pdf]

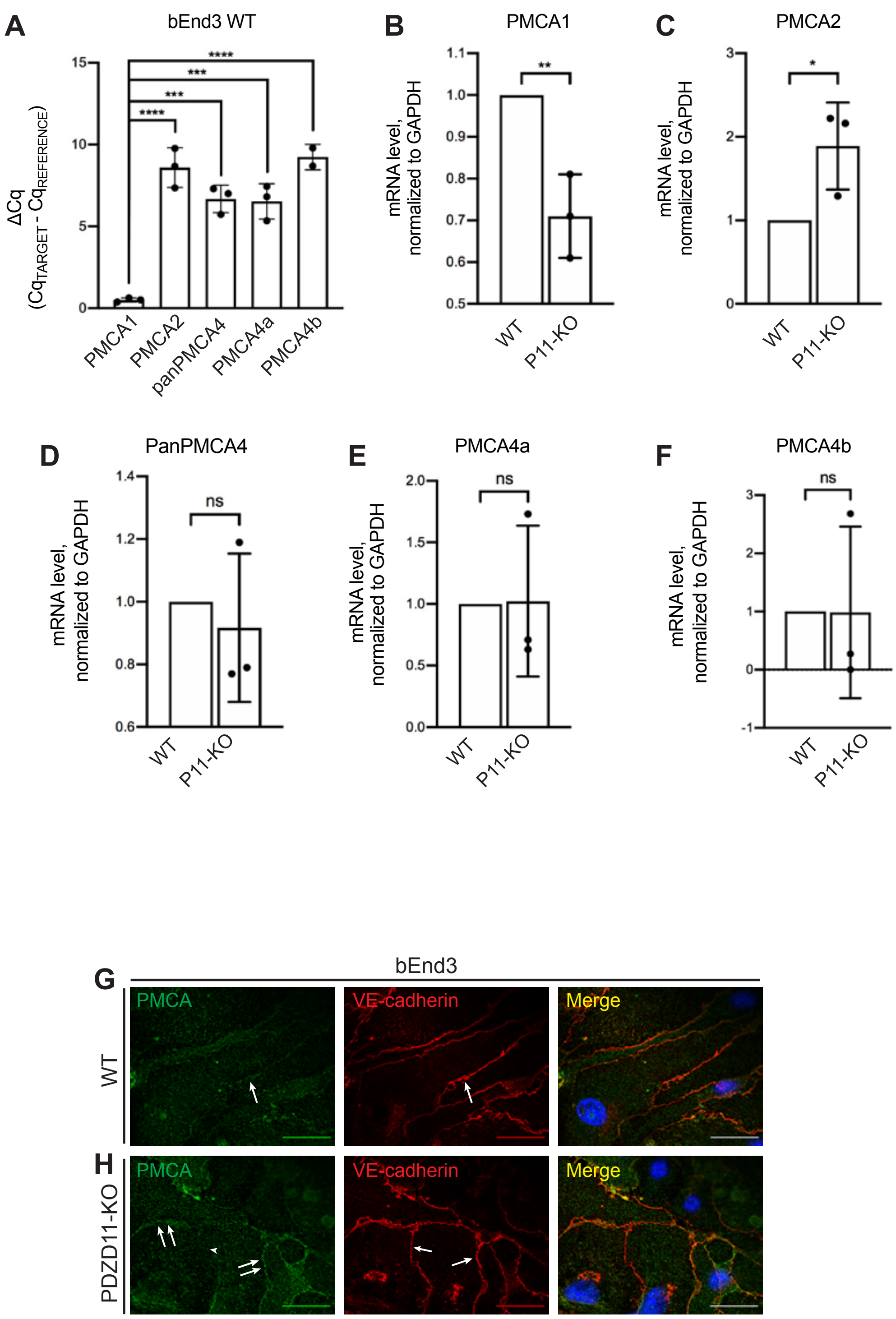

A
bEnd3 WT
ΔCq
(CqTARGET - CqREFERENCE)
****
***
***
****
PMCA1
PMCA2
panPMCA4
PMCA4a
PMCA4b
B
PMCA1
**
mRNA level,
normalized to GAPDH
WT
P11-KO
C
PMCA2
*
D
PanPMCA4
ns
E
PMCA4a
ns
F
PMCA4b
ns
G
bEnd3
WT
PMCA
VE-cadherin
Merge
H
PDZD11-KO

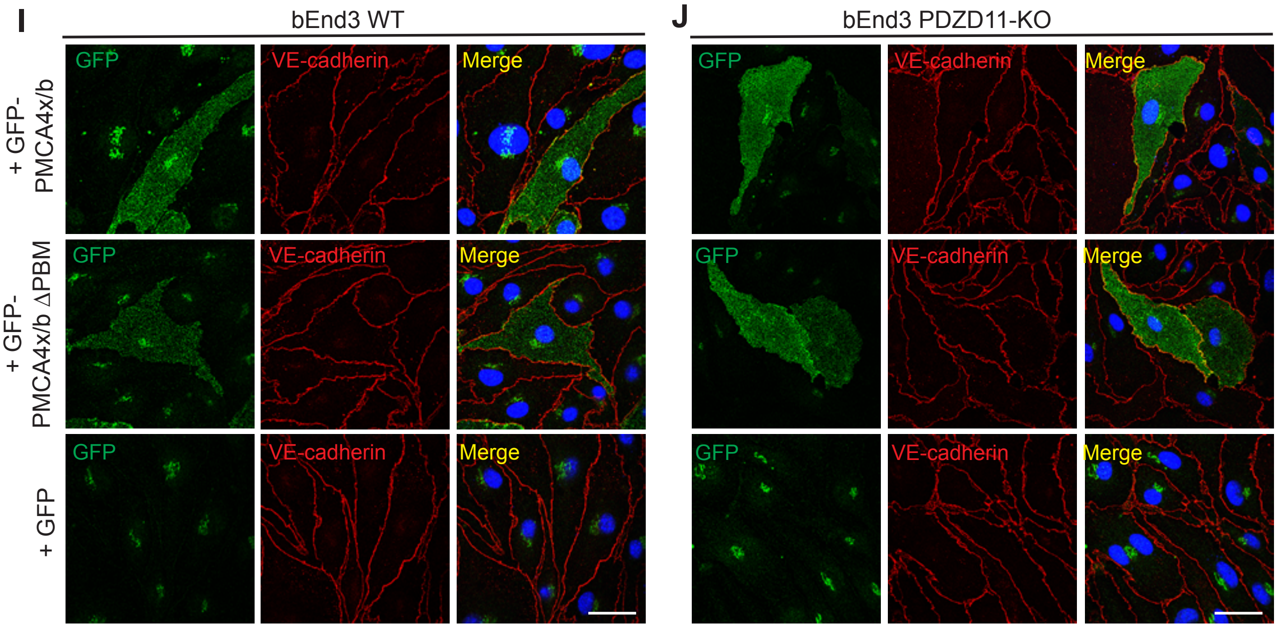

Figure S3 (Related to Figure 7). mRNA levels of PMCA isoforms, and IF microscopy localization of endogenous panPMCA and exogenous PMCA4x/b and PMCA4x/bDPBM in WT and PDZD11-KO bEnd3 cells.

(A-F) (A) mRNA expression profiles of PMCA isoforms in bEnd3 WT cells (A), and comparison between WT vs PDZD11-KO bEnd3 cells (B-F) (see legend Figure S1). Dots show replicates (n=34), and bars represent mean and SD. One-way ANOVA with post hoc Sidak's multiple comparisons test (*p<0.05, **p<0.005, ***p<0.0005 ****p<0.0001, ns: not significant).

(G-H) IF microscopy analysisof endogenous PMCA (panPMCA antibody) in WT (G) and PDZD11-KO (H) bEnd3 cells. VE-cadherin was used as a reference for junctions. Arrows indicate labeling at cxell periphery/junction, double arrows iundicate increased labeling, arrowhead reduced/undetectable labeling. Note that no consistent increase/decrease PMCA labeling at the cell periphery was detected in PDZD11-KO cells. Scale bar= 20 µm.

(I-J) IF microscopy analysis of exogenous GFP-PMCA4x/b (top panels), GFP-PMCA4x/bDPBM (middle panels) and GFP (bottom panels) in WT (I) and PDZD11-KO (J) bEnd3 cells. Scale bar= 20 µm.
